# High-Frequency Repetitive Magnetic Stimulation Activates Bactericidal Activity of Macrophages via Modulation of p62/Keap1/Nrf2 and p38 MAPK Pathways

**DOI:** 10.3390/antiox12091695

**Published:** 2023-08-30

**Authors:** Therese B. Deramaudt, Ahmad Chehaitly, Théo Charrière, Julie Arnaud, Marcel Bonay

**Affiliations:** 1 U1179 INSERM, END-ICAP, UFR des Sciences de la Santé-Simone Veil, Université de Versailles Saint-Quentin-en-Yvelines, 78180 Montigny-le-Bretonneux, Francemarcel.bonay@aphp.fr (M.B.); 2 Service de Physiologie-Explorations Fonctionnelles, Hôpital Ambroise Paré, Assistance Publique-Hôpitaux de Paris, 92100 Boulogne-Billancourt, France

**Keywords:** Nrf2, THP-1-derived macrophages, alveolar macrophages, repetitive magnetic stimulation, p38 MAPK, p62, Nrf2 knockout mice, Keap1, *Staphylococcus aureus*

## Abstract

The effects of repetitive magnetic stimulation (rMS) have predominantly been studied in excitable cells, with limited research in non-excitable cells. This study aimed to investigate the impact of rMS on macrophages, which are crucial cells in the innate immune defense. THP-1-derived macrophages subjected to a 5 min session of 10 Hz rMS exhibited increased Nrf2 activation and decreased Keap1 expression. We found that activation of the Nrf2 signaling pathway relied on rMS-induced phosphorylation of p62. Notably, rMS reduced the intracellular survival of *Staphylococcus aureus* in macrophages. Silencing Nrf2 using siRNA in THP-1-derived macrophages or utilizing Nrf2 knockout in alveolar macrophages abolished this effect. Additionally, rMS attenuated the expression of IL-1β and TNF-α inflammatory genes by *S. aureus* and inhibited p38 MAPK activation. These findings highlight the capacity of rMS to activate the non-canonical Nrf2 pathway, modulate macrophage function, and enhance the host’s defense against bacterial infection.

## 1. Introduction

Macrophages, as sentinel cells of the innate immune system, play a crucial role in innate immunity by recognizing and eliminating foreign invaders. Their ability to rapidly respond to microbial challenges and modulate immune responses is essential for host defense [1]. Macrophages migrate to sites of aggression, phagocytize external particles including microorganisms and cell debris, and initiate rapid responses for their elimination. While phagocytosis is essential for protection against bacterial infection, it can also serve as a pathway for bacteria, such as *Staphylococcus aureus*, to enter macrophages. These intracellular pathogens have developed evasion mechanisms to survive, escape, and disseminate within the host.

*S. aureus* is a Gram-positive bacterium that has colonized the human skin and mucous membranes. A versatile and commensal opportunistic pathogen, *S. aureus* can cause various infections in humans, including skin and soft tissue infections, pneumonia, osteomyelitis, and endocarditis [2]. Studies in a murine model of airway infection have highlighted the active contribution of macrophages in clearing methicillin-resistant *S. aureus* from the lungs. Depletion of alveolar macrophages in this mouse model resulted in increased mortality [3]. The prevalence of antibiotic-resistant *S. aureus* strains presents significant challenges, leading to higher morbidity, mortality, and healthcare costs worldwide [4]. Therefore, it is crucial to develop novel therapeutic strategies that can suppress the intracellular load of *S. aureus* while minimizing potential side effects to the host. *S. aureus* has the ability to manipulate the MAPK signaling pathway and modulate host cell responses for its benefit. Activation of specific MAPK pathways, including ERK, JNK and p38, has been implicated in promoting cell processes such as internalization, apoptosis and the expression of inflammatory cytokines and chemokines [5,6,7,8].

Our previous studies have confirmed the significant role of Nrf2 activity in influencing macrophage bactericidal function and immune responses [5,9]. Nrf2, a master transcriptional factor, acts as a key regulator in the adaptive responses to oxidative stress by regulating the expression of genes involved in antioxidant and cytoprotective mechanisms. Activation of Nrf2 leads to the transcriptional upregulation of cytoprotective genes that encode molecules involved in detoxifying, antioxidant and anti-inflammatory pathways [10].

Interestingly, Nrf2 can be activated through both canonical and non-canonical mechanisms [11]. The canonical mechanism involves the activation of Nrf2 through the Nrf2/Keap1-Cullin3-E3 ubiquitin ligase complex. Under homeostatic conditions, Nrf2 is sequestered in the cytoplasm by Keap1, which promotes its ubiquitination and subsequent degradation by the proteasome. However, in response to oxidative stress or electrophilic insults, Keap1 undergoes a conformational change, leading to the release of Nrf2 and its translocation into the nucleus. Nuclear Nrf2 then heterodimerizes with small Maf proteins to form a cofactor complex that binds to antioxidant responsive element (ARE) sequences, resulting in the upregulation of genes coding for proteins involved in antioxidant and anti-inflammatory responses.

Several non-canonical mechanisms of Nrf2 activation have also been described and involved direct interactions between Keap1 and various proteins, such as the scaffold protein p62/SQSTM1 [12], the dipeptidyl peptidase III enzyme [13], the nuclear protein prothymosin α [14], and the nuclear protein partner and localizer of BRCA2 (PALB2) [15]. These interactions promote the nuclear accumulation of Nrf2 and the transcriptional regulation of Nrf2-targeted genes.

Repetitive magnetic stimulation (rMS) is a non-invasive technique primarily used in the treatment of neurological disorders, with potential therapeutic applications in neuroregeneration and wound healing [16,17]. While the cellular responses elicited by rMS have been extensively documented in excitable cells, limited studies have investigated the impact of rMS on non-excitable cells. A recent report has highlighted the ability of microglia, the resident immune cells of the central nervous system, to modulate neural excitability and plasticity through the release of cytokines following high-frequency repetitive transcranial magnetic stimulation (rTMS) in mice [18]. Additionally, high-frequency rTMS treatment of bone mesenchymal stromal cells has been shown to induce autophagy through the NMDAR-Ca^2+^-ERK-mTOR signaling pathway [19]. Still, it is important to note that the effects of rMS on cellular processes can vary depending on the cell type and are influenced by various stimulatory parameters, including the intensity, frequency, and duration of the magnetic pulses. Nonetheless, the molecular and cellular mechanisms underlying the specific effects of rMS on cell types outside the nervous system remain poorly understood.

This study aims to explore the effect of rMS on the intracellular survival of *S. aureus* in THP-1-derived macrophages and murine primary alveolar macrophages. We demonstrate that the induction of the Nrf2/Keap1/p62 signaling pathway by rMS is critical for controlling the intracellular load of *S. aureus*. Furthermore, we established that rMS treatment represses the *S. aureus*-mediated activation of p38 MAPK. To the best of our knowledge, this is the first study to provide insights into the molecular and cellular mechanisms of a single 5 min session of high-frequency rMS in in vitro THP-1-derived macrophages and ex vivo murine primary alveolar macrophages.

## 2. Materials and Methods

### 2.1. Antibodies and Reagents

A Maxima First Strand cDNA synthesis kit, Lipofectamine^®^ LTX & PLUS™ Reagent, Trizol, CellROX™ Green Oxidative Stress Reagent, Fluoromount-G^TM^ (EMS), RIPA Lysis and Extraction Buffer, sodium pyruvate, Pierce protease and phosphatase inhibitor cocktail, SYTO9^TM^ dye from the LIVE/DEAD BacLight bacterial counting kit, anti-phospho-p62 antibody (pS349, Invitrogen), 3-(4,5-dimethylthiazol-2-yl)-2,5-diphenyltetrazolium bromide (MTT) cell viability assay kit (Biotium Inc.), secondary antibodies donkey anti-mouse Alexa Fluor 488, donkey anti-rabbit Alexa Fluor 594, and an iBind Western blot system were purchased from Fisher Scientific (Illkirch, France). A nuclear extraction kit, phorbol 12-myristate 13-acetate (PMA), dimethyl sulfoxide (DMSO) ≥ 99.7%, β-mercaptoethanol, paraformaldehyde, H_2_O_2_ 30% in H_2_O, rabbit anti-phosphorylated ERK (pT185/pY187) antibody, rabbit anti-ERK antibody, mouse anti-phospho-p38 (pT180/pY182) antibody, rabbit-anti-p62 antibody, rabbit anti-Keap1 antibody, and mouse anti-GAPDH antibody were obtained from Merck (Saint-Quentin-Fallavier, France). iTaq SYBRgreen qPCR Supermix, a DC protein assay kit, and 4–20% mini-Protean precast protein gels were purchased from BioRad (Marnes-la-Coquette, France). RPMI (Roswell Park Memorial Institute) 1640 culture medium, phosphate-buffered saline (PBS), gentamicin, penicillin/streptomycin mixture, Hoechst 33342, and heat-inactivated fetal bovine serum were obtained from Eurobio Scientific (Les Ulis, France). CO_2_ ≥ 99.99% was purchased from Messer (France). Annexin V-fluorescein isothiocyanate (FITC) apoptosis detection reagent, mouse anti-HO-1 antibody, rabbit anti-NQO1 antibody, and rabbit anti-Lamin A/C antibody were purchased from Abcam (Paris, France). Mouse anti-phospho-JNK (pT183/pY185) antibody, mouse anti-JNK antibody, mouse anti-p38 antibody were acquired from BD Biosciences (Le Pont de Claix, France). Anti-Nrf2 antibody was obtained from ProteinTech (Manchester, UK). Tryptic soy broth (TSB) and agar-supplemented TSB (TSB agar) were obtained from Conda laboratories (Dutscher, Bernolsheim, France). IRDye^®^ 680CW goat anti-mouse IgG, and IRDye^®^ 800CW Goat anti-Rabbit IgG secondary antibodies were purchased from LI-COR^®^ Biosciences (Bad Homburg, Germany).

### 2.2. Repetitive Magnetic Stimulation (rMS)

Repetitive magnetic stimulation was delivered using a B65 refrigerated butterfly coil connected to a MagPro R30 magnetic stimulator (Magventure, Denmark) purchased from Mag2Health (Villennes-sur-Seine, France). Cells were placed on the cool coil and were subjected to one session of 10 series of 100 biphasic pulses with a 25 s interval between series, resulting in a total of 1000 pulses (10 Hz at 80% of maximum output). In parallel, control cells were exposed to the same environment for the same duration as the stimulated cells but without stimulation. rMS at 5 Hz and 15 Hz were also tested and showed no effect on HO-1 and NQO1 mRNA expressions (Supplementary Figure S1).

### 2.3. Bacterial Strain, Growth Culture, and Fluorescence Labeling

The Gram-positive bacteria *Staphylococcus aureus* strain (ATCC 25923) was grown aerobically in Trypticase soy broth to the optical density of 1 at 37 °C under agitation. Glycerol stocks of *S. aureus* were prepared and aliquoted. When required, frozen stocks were thawed and bacteria were diluted in sterile PBS at the appropriate concentration.

For phagocytosis assay, *S. aureus* was incubated with SYTO9 dye for 15 min in the dark following the manufacturer’s instructions. For immunofluorescence microscopy, *S. aureus* was incubated with 10 µg/mL Hoechst 33342 in the dark. After 1 h, Hoechst 33342-stained bacteria were washed twice with PBS and resuspended in PBS.

### 2.4. Cell Culture, Cell Differentiation, and Cell Transfection

Human monocytic THP-1 cell line (ATCC^®^ TIB-202™) was maintained in RPMI 1640 Glutabio medium supplemented with 10% heat-inactivated fetal bovine serum, 10 mM HEPES buffer, 1 mM sodium pyruvate, and 50 µM β-mercaptoethanol in a humidified atmosphere at 37 °C and 5% CO_2_. Terminal differentiation of THP-1 to macrophages was obtained by rinsing cells twice with PBS prior to incubation with 50 nM PMA in β-mercaptoethanol-free complete RPMI 1640 medium for 48 h.

*S. aureus* was added to the cell culture at a multiplicity of infection (MOI) of 10. For experiments requiring an incubation time longer than 3 h, 10 µg/mL gentamicin was added to the cell culture medium to inhibit growth of extracellular bacteria.

In order to knockdown Nrf2, THP-1-derived macrophages were transfected with a pre-designed siRNA targeting Nrf2 (s9492; ThermoFisher Scientific) or a universal negative control siRNA (1027310; Qiagen) using Lipofectamine LTX following the manufacturer’s recommended protocol.

### 2.5. Animals and Isolation of Primary Mouse Alveolar Macrophages

Breeding of the C57BL/6J Nrf2 knockout (Nrf2^−/−^) mouse strain, provided by Dr Yamamoto (Tohoku University) and purchased from Riken BRC [20], was conducted according to National and European legislation and institutional guidelines for the care and use of laboratory animals approved by the Animal Ethics Committee CEEA47 PELVIPHARM (2Care animal facility, University of Versailles Saint-Quentin-en-Yvelines, UFR Santé Simone Veil) and the Ministry of Higher Education, Research and Innovation APAFIS#8785-201610191723731v3. Mice were maintained in a standard 12 h light/12 h dark cycle with access to food and water ad libitum.

Bronchoalveolar lavage was performed following carbon dioxide euthanasia of mice and alveolar macrophages were collected via repeated lavages with 1 mL ice-cold PBS. After cell resuspension, total cell count was obtained using Countess automated Cell counter (Invitrogen, ThermoFisher Scientific, Waltham, MA, USA). Primary alveolar macrophages were maintained in complete RPMI 1640 medium complemented with penicillin/streptomycin mixture overnight. The antibiotics were removed prior to performing ex vivo experiments by washing cells twice with PBS, followed by the addition of antibiotics-free complete RPMI 1640 medium.

### 2.6. Bacteria Intracellular Survival Assay

THP-1-derived macrophages, seeded in 24-well plates at 2.5 × 10^5^ cells/well, were treated with rMS for 3 h prior to infection with *S. aureus* at an MOI of 10. Gentamicin was added to the cell culture medium 1 h after infection to inhibit growth of extracellular bacteria, and macrophages were incubated for a total of 24 h before the colony-forming unit (CFU) assay. Briefly, macrophages were washed twice with ice-cold PBS followed by 20 min incubation in 1 mL ice-cold sterile water to lyse cells. Numeration of intracellular bacteria was obtained by plating 5-fold serial dilutions on TSB solidified with 1.5% agar and incubating at 37 °C for 24 h.

### 2.7. Subcellular Fractionation and Immunoblotting

For the subcellular fractionation, a nuclear extraction kit was used according to the manufacturer’s protocol. Briefly, following treatment of THP-1-derived macrophages, 3 × 10^6^ cells per condition were collected and washed with PBS. After centrifugation, cell pellets were incubated for 15 min with ice-cold cytoplasmic lysis buffer supplemented with a protease and phosphatase inhibitor cocktail. Cell membranes were then disrupted via repeated passages through a 27G needle. After 20 min centrifugation at 8000× *g* at 4 °C, the cytoplasmic fractions were collected and the remaining cell pellets were resuspended in ice-cold nuclear extraction buffer supplemented with protease and phosphatase inhibitor cocktail. Nuclear membranes were disrupted via repeated passages through a 27G needle, followed by incubation of the nuclear suspensions at 4 °C on an orbital shaker. After 1 h, nuclear fractions were collected by centrifugation at 16,000× *g* for 5 min at 4 °C.

For extraction of total protein lysates, 1 × 10^6^ THP-1-derived macrophages were rinsed once with PBS and lysed in ice-cold RIPA buffer supplemented with a protease and phosphatase inhibitor cocktail. After 30 min incubation on ice, cell lysates were centrifuged at 12,000× *g* for 5 min at 4 °C. The supernatants containing protein extracts were collected.

Protein concentrations for total protein extracts and subcellular fractions were measured using a DC protein assay kit. Protein extracts were resolved by SDS-PAGE and transferred to a polyvinylidene difluoride membrane (Immobilon-FL, Merck, Darmstadt, Germany). Immunoblotting was performed using the iBind flex western system according to the manufacturer’s instructions. Briefly, primary antibodies targeting Nrf2, HO-1, NQO1, p62, phospho-p62, p38, phospho-p38, ERK, phospho-ERK, JNK, phospho-JNK, GAPDH, and secondary antibodies, IRDye680RD and IRDye800RD, were diluted in iBind Flex FD solution. Fluorescence signals were acquired using Odyssey CLx imaging system (LI-COR) and densitometric analysis was achieved using Image Studio Lite v4.0.

### 2.8. Total RNA Extraction and Real-Time Quantitative PCR Analysis

Total RNA was isolated from treated THP-1-derived macrophages using Trizol reagent and chloroform extraction technique following the manufacturer’s instructions. RNA concentrations were determined using the NanoPhotometer^®^ N120 (Implen; München, Germany). First, 1 µg of total RNA was reverse transcribed to cDNA using a Maxima First strand cDNA synthesis kit. Quantitative analysis was achieved using real-time quantitative PCR (RT-qPCR), with each cDNA sample performed in triplicate. qPCR was realized using the BioRad CFX384 Touch Real-Time PCR Detection system and iTaq SYBRgreen qPCR mix. Table 1 lists the specific primers used for qPCR, Nrf2, HO-1, NQO1, IL-6, IL-1β, TNF-α, and 18S rRNA, and synthetized by Eurogentec (Seraing, Belgium). The cycling parameters for qPCR were 95 °C for 3 min, 40 cycles of 95 °C for 5 s and 60 °C for 20 s, with a melting curve from 65 °C to 95 °C. The cycle threshold (Ct) values of each target genes were first normalized to that of the reference gene 18S rRNA (ΔCt) then the final values (ΔΔCt values) were expressed as folds of control. Data were analyzed on the BioRad CFX manager 3.1 using the ΔΔCt method.

### 2.9. Analysis of Cell Phagocytosis and Cell Apoptosis via Flow Cytometry

For the assessment of cell phagocytosis, 1 × 10^6^ cells/well THP-1-derived macrophages were treated with rMS 3 h prior to infection with SYTO9-labeled *S. aureus*. After 1 h infection, macrophages were scraped, washed with PBS, and collected for flow cytometry analysis.

To assess cell apoptosis 24 h after rMS treatment, 1 × 10^6^ cells/well THP-1-derived macrophages were stained for 10 min in the dark with Annexin V-FITC reagent. Non-adherent and adherent cells were collected and after 3 washes with PBS, cells were analyzed via flow cytometry.

All flow cytometric analysis was immediately realized on the BD LSRFortessa^TM^ using FACSDiva 7.0 software (BD Biosciences, Franklin Lakes, NJ, USA).

### 2.10. Quantification of Intracellular ROS Production

Intracellular ROS was measured in live cells using the CellROX^TM^ green fluorogenic probe. Briefly, 2.5 × 10^5^ THP-1-derived macrophages grown on 12 mm-diameter coverslips were treated with rMS. After 6 h incubation, CellROX reagent was added to the cell culture medium. After an additional 30 min, cells were washed with PBS, then fixed in 4% paraformaldehyde (PFA) in PBS. Nuclei were counterstained with 4′,6-diamidino-2-phenylindole (DAPI), and macrophages were mounted on glass slides using Fluoromount g aqueous mounting medium. Positive control for ROS production consisted of treatment of macrophages with H_2_O_2_. Confocal fluorescent images were taken using Leica SP8 confocal microscope (Leica Microsystems, Wetzlar, Germany) and quantitative analysis of fluorescent signals was performed for 7 fields per treatment using Image J v1.53k (National Institutes of Health, Rockville, MD, USA).

### 2.11. Cell Viability Assay

Cell viability was assessed using the MTT assay. Briefly, 5 × 10^4^ cells/well THP-1-derived macrophages seeded in a 96-well plate were treated for 24 h before measuring the cellular metabolic activity following the manufacturer’s protocol. Absorbance values were obtained at 550 nm and 600 nm using the FLUOstar Omega microplate reader (BMG Labtech).

### 2.12. Immunofluorescence Microscopy

THP-1-derived macrophages were obtained by seeding 5 × 10^4^ THP-1 cells on glass coverslips in 24-well plates followed by differentiation with PMA for 48 h. After rMS treatment and/or *S. aureus* infection, macrophages were fixed in 4% PFA in PBS, permeabilized with 0.1% Triton X-100 for 5 min, blocked with 1% BSA in PBS for 1 h, and then incubated overnight at 4 °C with the primary antibodies targeting p62 or phospho-p38 diluted in 1% BSA in PBS. After being washed with PBS, cells were incubated with secondary antibodies diluted in 1% BSA for 1 h at room temperature. Nuclei were counterstained with Hoechst 33342. Coverslips were mounted on slides using Fluoromount-G. Confocal images of p62/SQSTM1 punctate structures were acquired using a Leica SP8 confocal microscope, 40× magnification. Quantitative analyses of fluorescent signals were performed on 10 fields per treatment using Image J v1.53k (National Institutes of Health, Bethesda, MD, USA).

### 2.13. Statistical Analyses

Values are presented as means ± standard errors of mean (SEM). The imaging flow cytometry results presented are means ± SEM of 3 independent experiments of 100,000 events. Statistical comparisons were performed using a two-tailed unpaired *t*-test for comparisons between 2 groups, or one-way analysis of variance with Tukey’s multiple group comparison test for multiple comparisons. Data analyses were performed with GraphPad Prism statistical software (v8.0.2). Differences were considered to be statistically significant at *p* < 0.05.

## 3. Results

### 3.1. High-Frequency rMS Activates Nrf2 in THP-1-Derived Macrophages

**Figure 1:**
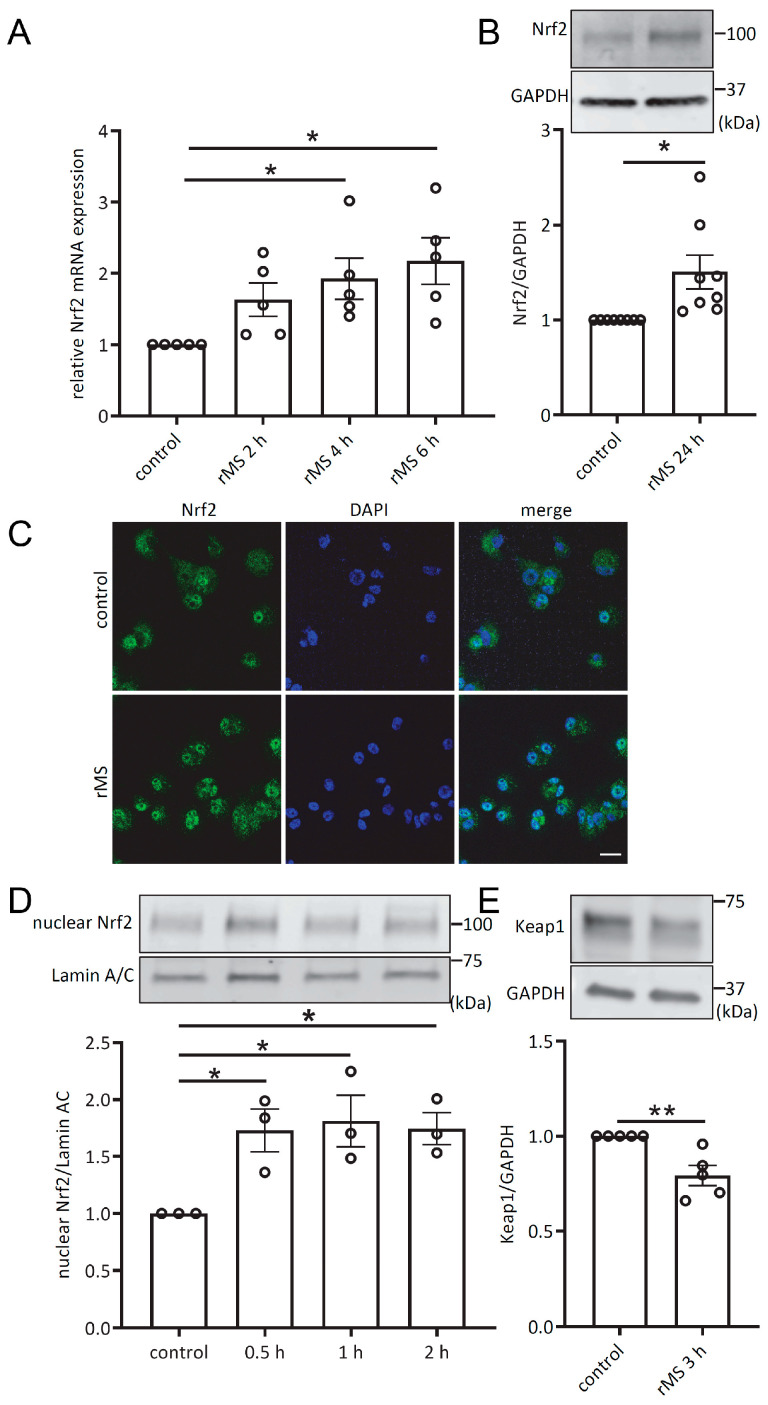
rMS activates Nrf2 in THP-1-derived macrophages. (**A**) THP-1-derived macrophages subjected to rMS treatment (10 Hz). Nrf2 mRNA expression was determined via RT-qPCR. Data were analyzed using a one-way ANOVA followed by Dunnett’s multiple comparisons. (**B**) Nrf2 protein expression was detected via Western blotting 24 h after rMS treatment and analyzed using densitometric analysis. Statistical analysis was performed using two-tailed unpaired *t*-test. (**C**) Immunofluorescence microscopy. Nuclear translocation of Nrf2 1 h after rMS treatment. THP-1-derived macrophages were immunostained with specific Nrf2 antibody; nuclei were conterstained with DAPI. Bars: 20 µm. (**D**) Nuclear Nrf2 protein expression was determined at the indicated time point using Western blotting and analyzed using densitometry analysis. Data are expressed as Nrf2 protein expression normalized to lamin A/C control values. Statistical analysis was performed using one-way ANOVA followed by Dunnett’s multiple comparisons. (**E**) Keap1 protein expression was detected 3 h after rMS treatment and analyzed using densitometric analysis. Statistical analysis was performed using two-tailed unpaired *t*-test. Data are shown as mean ± SEM * *p* < 0.05, ** *p* < 0.01.

In order to determine the effect of repetitive magnetic stimulation on macrophages, THP-1-derived macrophages were treated with rMS. After the indicated incubation time, RT-qPCR analysis showed a significant increase in Nrf2 transcriptional expression at 4 h and 6 h after rMS treatment (Figure 1A). At 24 h after rMS treatment, Nrf2 protein expression was measured via Western blot, and was found to be significantly increased compared to control (Figure 1B). Confocal images were taken of THP-1-derived macrophages treated with rMS and immunolabeled with a specific antibody against Nrf2, with the nucleus counterstained with DAPI. An increasing amount of Nrf2 localized within the nucleus was observed compared to control (Figure 1C). To quantify the amount of Nrf2 localized in the nucleus, nuclear extracts were examined via Western blot. A significant increase in nuclear Nrf2 was observed as early as 30 min after rMS treatment (Figure 1D). Moreover, we found that the expression of Keap1 protein was significantly decreased in THP-1-derived macrophages 3 h after rMS treatment (Figure 1E). These results suggest that rMS treatment is able to modulate the activation of Nrf2 and Keap1 by affecting their expression and/or localization in macrophages.

### 3.2. rMS Activates Nrf2 Signaling Pathway

**Figure 2:**
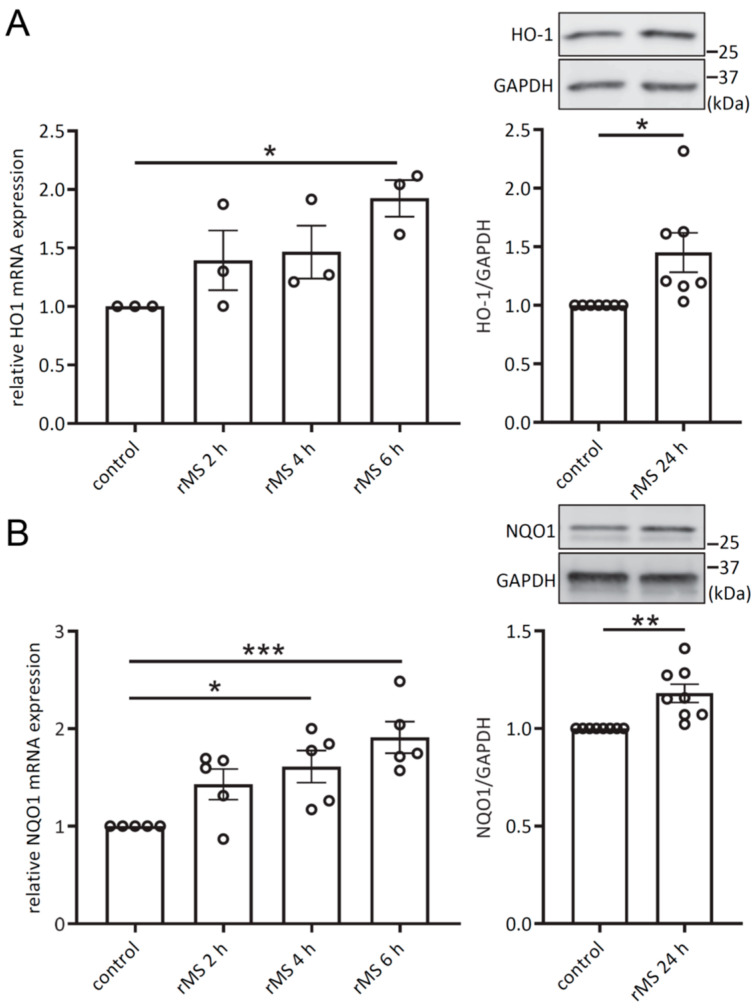
rMS increases HO-1 and NQO1 mRNA and protein expression levels. (**A**) Transcriptional expression of HO-1 at the indicated time points was determined via SYBRgreen RT-qPCR. Data shown are mean ± SEM from independent experiments. Statistical analyses were performed using one-way ANOVA followed by Dunnett’s multiple comparisons. HO-1 protein expression was determined at 24 h after rMS treatment. (**B**) NQO1 mRNA and protein expressions. * *p* < 0.05; ** *p* < 0.01; *** *p* < 0.005.

To determine whether the nuclear translocation of Nrf2 leads to the activation of the Nrf2 signaling pathway, we examined the transcriptional and protein expressions of HO-1 and NQO1, two downstream targets of Nrf2. RT-qPCR analyses were performed on total mRNA extracted from THP-1-derived macrophages at 2, 4 and 6 h after rMS treatment. RT-qPCR showed a significant increase in HO-1 mRNA expression at 6 h (Figure 2A). Immunoblotting analysis conducted on protein lysates extracted from THP-1-derived macrophages 24 h after rMS treatment revealed a significant increase in HO-1 protein expression (Figure 2A). Additionally, a significant increase in NQO1 mRNA expression level was observed at 4 h and 6 h after rMS treatment and NQO1 protein expression level exhibited a significant increase at 24 h after rMS treatment (Figure 2B). These results confirmed the activation of the Nrf2 signaling pathway by rMS.

### 3.3. rMS Increased Phosphorylated p62 and Total p62 Protein Expressions

**Figure 3:**
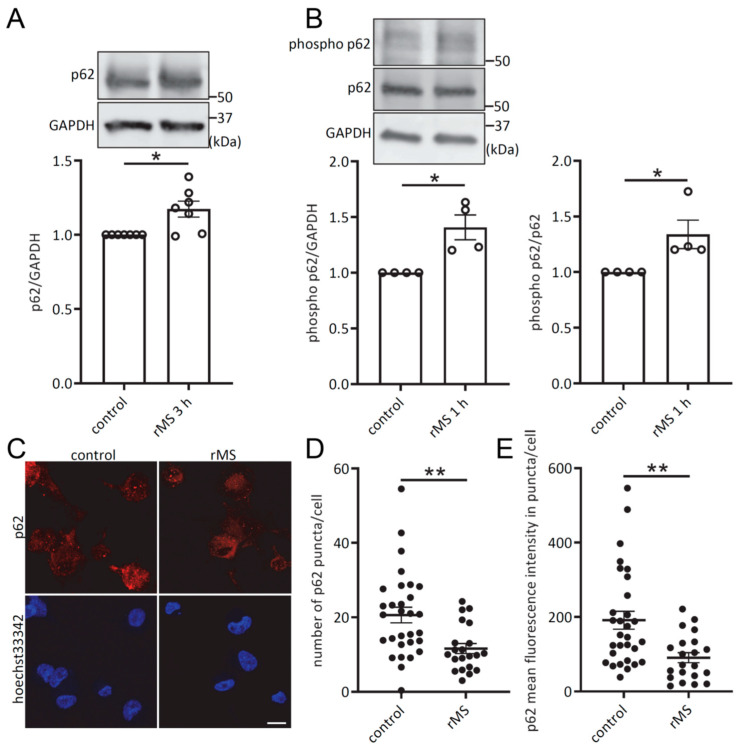
rMS activates p62 and increases p62 protein expression level. (**A**,**B**) p62 and phosphorylated p62 protein expressions were determined via Western blotting 3 h and 1 h after rMS treatment, respectively. (**C**) Confocal microscopy images of THP-1-derived macrophages treated with rMS. Immunofluorescence labeling with p62 and counterstained with Hoechst33342 was performed. Bars: 10 µm. (**D**,**E**) Quantitative analysis of p62 puncta and p62 fluorescence intensity in the puncta using Image J. Mean ± SEM were obtained from analysis of n = 100 cells from 2 independent experiments. * *p* < 0.05, ** *p* < 0.01. Statistical analyses were performed using two-tailed unpaired *t*-test.

Next, we hypothesized that the observed decrease in Keap1 expression and increase in Nrf2 expression levels resulted from rMS modulation of p62 phosphorylation and p62 expression levels. Total protein extracts were collected from THP-1-derived macrophages 1 h after rMS treatment for phosphorylated p62 immunoblotting analysis and 3 h after rMS treatment for p62 immunoblotting analysis. Data analysis showed a significant increase in p62 protein expression, as well as a significant increase in phosphorylated p62 (Figure 3A,B). Furthermore, confocal images were taken from THP-1-derived macrophages treated with rMS for 3 h. The macrophages were then immunolabeled with a specific p62 antibody, and the nucleus was counterstained with Hoechst33342 (Figure 3C). Using Image J software v1.53k, the number of p62 puncta and the fluorescence intensity of p62 in the puncta were quantified. Our data showed a decrease in the number of p62 puncta per cell in rMS-treated macrophages, along with a decrease in p62 fluorescent intensity within the puncta of each cell (Figure 3D,E). These results suggest that rMS induces the phosphorylation of p62, which enables the binding of phosphorylated p62 with Keap1, and consequently allows the released Nrf2 to translocate into the nucleus. The phosphorylated p62-Keap1 complex subsequently undergoes p62-dependent autophagy degradation [21].

### 3.4. Effects of rMS on Cell Viability, Oxidative Stress, Inflammation, and Macrophage Bactericidal Function

**Figure 4:**
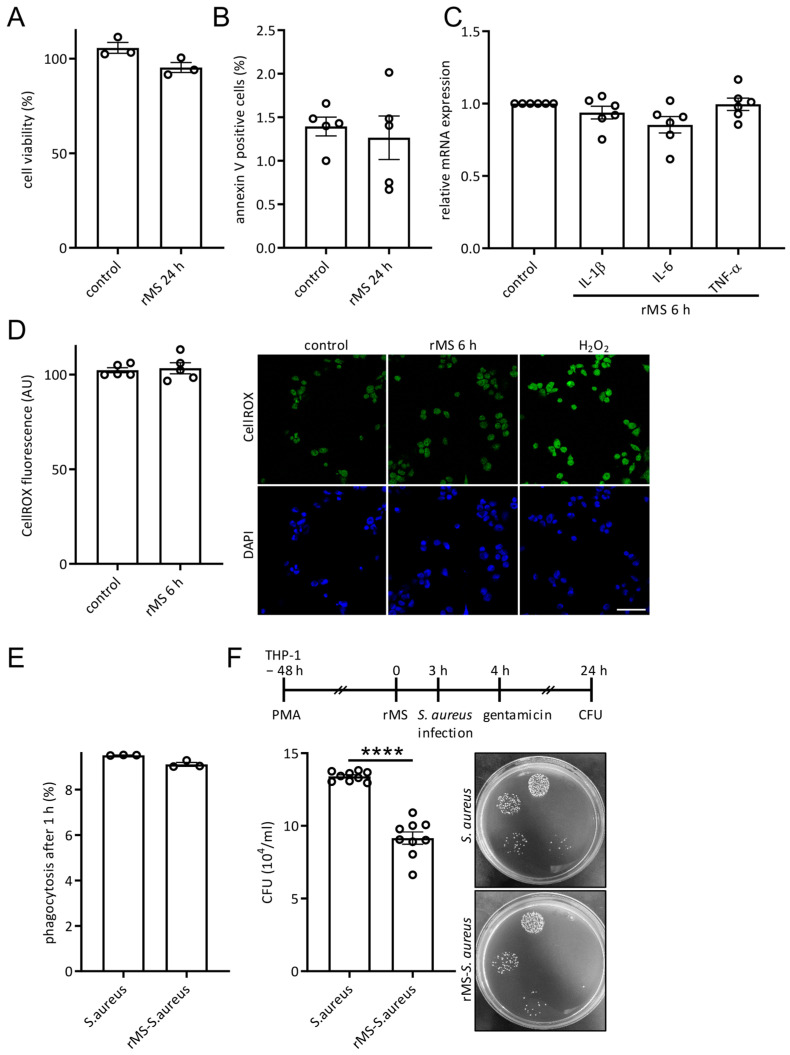
Effects of rMS on cell viability, oxidative stress, inflammation, and macrophage bactericidal function. (**A**) Cell viability measured via MTT assay 24 h after rMS treatment. (**B**) Cell apoptosis was determined using the Annexin V staining. (**C**) Inflammation was determined by quantifying IL-6, IL-1β, and TNF-α mRNA expressions 6 h after rMS treatment using RT-qPCR. (**D**) Oxidative stress was measured using a CellROX fluorogenic probe in live cells to detect ROS. Quantification of CellROX green fluorescence involved Image J. H_2_O_2_-treated cells were used as positive control. Scale bar: 50 µm. (**E**) THP-1-derived macrophages were infected with SYTO9-labeled *S. aureus*. After 1 h infection, phagocytosis was determined via FACS. (**F**) *S. aureus* intracellular survival using CFU assay. Three hours after rMS treatment, THP-1-derived macrophages were infected with *S. aureus*. Extracellular bacteria were eliminated via addition of gentamicin 1 h after infection. Cells were incubated for 24 h and CFU was determined. Data are mean ± SEM. Statistical analysis was performed using two-tailed unpaired *t*-test **** *p* < 0.0001.

To further investigate the potential impact of rMS treatment on cellular processes, THP-1-derived macrophages were subjected to rMS and incubated for the indicated time before analysis. Firstly, we assessed the effect of rMS on cell viability using the MTT assay and cell apoptosis by staining with Annexin V. Flow cytometry analysis indicated that rMS treatment of THP-1-derived macrophages had no significant impact on cell viability or cell apoptosis (Figure 4A,B). We also performed RT-qPCR on mRNA extracted from THP-1-derived macrophages 6 h after rMS treatment to evaluate the transcriptional expression of genes coding for the inflammatory markers IL-1β, IL-6, and TNF-α. No significant changes were observed in their mRNA expressions (Figure 4C). Next, we investigated whether rMS affected oxidative stress by staining rMS-treated THP-1-derived macrophages with CellROX fluorogenic probe, which detected ROS in live cells. Data analysis showed no significant effect of rMS on oxidative stress (Figure 4D). We then examined the impact of rMS on macrophage phagocytic activity. THP-1-derived macrophages were treated with rMS and incubated for 3 h prior to infection with SYTO9-labeled *S. aureus* for 1 h. Flow cytometry analysis revealed no significant difference in macrophage phagocytosis activity after rMS as compared to control (Figure 4E). Furthermore, we assessed the bactericidal activity of rMS-treated macrophages by determining the intracellular survival of *S. aureus* 24 h after infection. Gentamicin was added to the cell culture medium 1 h after infection to eliminate extracellular bacteria. CFU analysis demonstrated a significant decrease in intracellular survival of *S. aureus* in rMS-treated THP-1-derived macrophages (Figure 4F). Collectively, these findings indicate that 5 min magnetic stimulation does not affect the cell viability, cell apoptosis, inflammation, or oxidative stress of THP-1-derived macrophages. Notably, rMS appears to impact the intracellular survival of *S. aureus*.

### 3.5. rMS-Mediated Decrease in S. aureus Intracellular Survival Is Nrf2 Dependent

**Figure 5:**
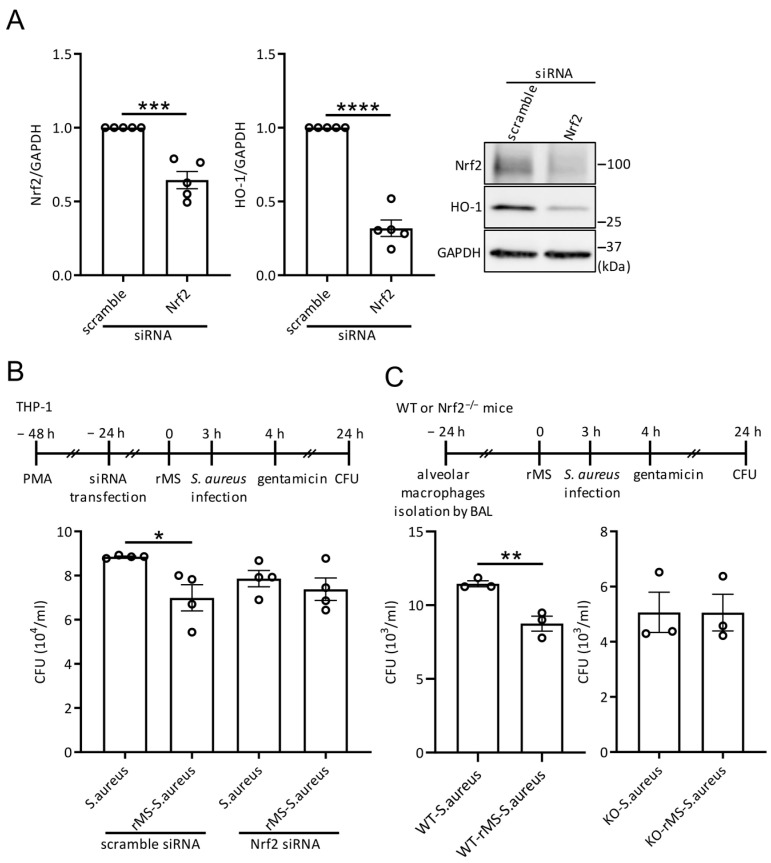
rMS-mediated decrease in *S. aureus* intracellular survival is Nrf2-dependent. (**A**) THP-1 derived macrophages were transfected with scramble or Nrf2-targeted siRNA 24 h prior to rMS treatment. Then, 24 h after rMS treatment, protein lysates were analyzed Nrf2 and HO-1 protein expressions were determined via Western blotting. Statistical analysis was performed using a two-tailed *t*-test *** *p* < 0.0003, **** *p* < 0.0001. (**B**) CFU with transfected THP-1 derived macrophages. (**C**) Primary alveolar macrophages were isolated from WT and Nrf2 knockout mice and used in bacteria intracellular survival assay. Data are means ± SEM. * *p* < 0.05, ** *p* < 0.01 as determined by one-way ANOVA with Tukey’s multiple comparisons.

In order to investigate whether the bactericidal activity of rMS-treated THP-1-derived macrophages resulted from Nrf2 activity, macrophages were transfected with either scrambled siRNA or siRNA specifically targeting Nrf2. Firstly, Western blot analysis confirmed the reduced expression level of Nrf2 and HO-1 proteins in Nrf2-targeted siRNA-transfected macrophages compared to those transfected with scrambled siRNA (Figure 5A). Next, we examined the intracellular survival of *S. aureus* in scrambled or Nrf2 siRNA-transfected macrophages. As expected, CFU assay demonstrated a significant decrease in intracellular survival of *S. aureus* in scrambled siRNA-transfected THP-1-derived macrophages treated with rMS compared to untreated cells. Interestingly, THP-1-derived macrophages transfected with Nrf2-targeted siRNA showed no rMS-mediated decrease in intracellular survival of *S. aureus* compared to untreated cells (Figure 5B).

Additionally, we evaluated the intracellular survival of *S. aureus* in primary alveolar macrophages isolated from Nrf2 knockout (KO) mice and their wild-type (WT) littermates (Figure 5C). Alveolar macrophages were isolated from bronchoaleolar lavages, cultured for 24 h, and then subjected to rMS treatment and *S. aureus* infection. Gentamicin was added to the cell culture medium 1 h after infection to inhibit the growth of extracellular bacteria. After 24 h, CFU numeration was determined. Quantitative analysis revealed that the rMS-mediated decrease in intracellular survival of *S. aureus* observed in alveolar macrophages from WT mice did not persist in rMS-treated alveolar macrophages isolated from Nrf2 KO mice. These results suggest the involvement of Nrf2 in the effect of rMS on the bactericidal activity of macrophages.

### 3.6. rMS Decreases IL-1β and TNF-α mRNAs Expression Levels and p62 Protein Expression Level in S. aureus-Infected THP-1-Derived Macrophages

**Figure 6:**
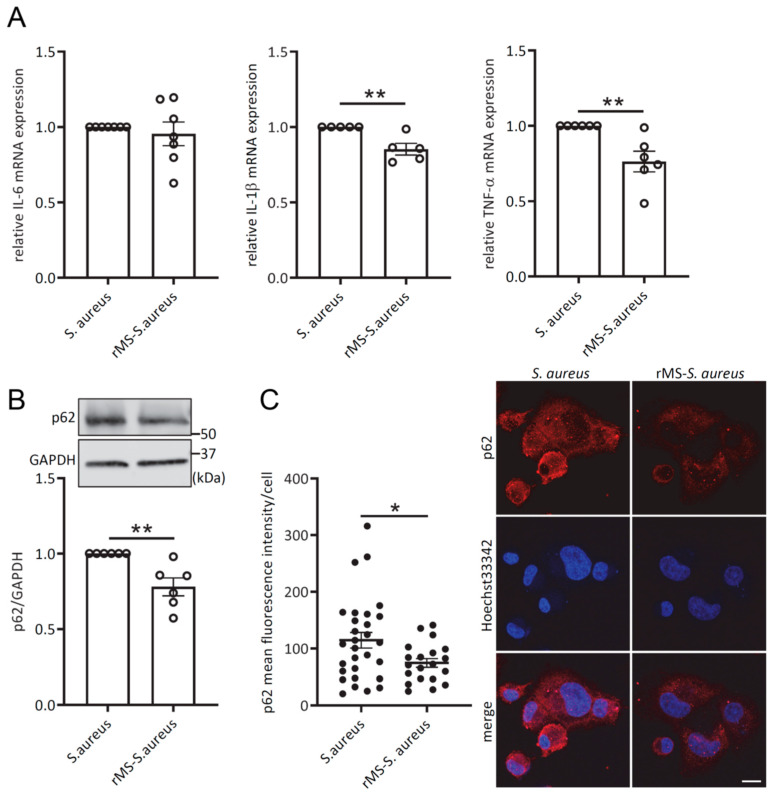
rMS decreases IL-1β and TNF-α mRNA expression levels and p62 protein levels in *S. aureus*-infected THP-1-derived macrophages. (**A**) rMS-treated THP-1-derived macrophages were infected with *S. aureus*. Total RNAs were isolated 3 h after infection. IL-6, IL-1β, and TNF-α mRNA expressions were quantified by RT-qPCR. (**B**) Immunoblot analysis. Protein lysates were extracted from THP-1-derived macrophages after 3 h treatment with rMS and infected with *S. aureus* for 3 h before cell lysis. Immunoblots using the indicated antibodies are representative of 6 independent experiments. (**C**) Immunofluorescence microscopy. THP-1-derived macrophages were treated with rMS then infected with Hoechst 33342-stained *S. aureus*. Cells were fixed with PFA 4% and were immunostained with p62 antibody. Nucleus was stained with Hoechst 33342. Bar scale: 10 µm. p62 mean fluorescence intensity in puncta by cell was quantified using Image J v1.53k. * *p* < 0.05, ** *p* < 0.01. Statistical analyses were performed using a two-tailed unpaired *t*-test.

Upon infection, *S. aureus* induces the expression of genes encoding pro-inflammatory cytokines, including IL-6, IL-1β, and TNF-α [5]. In THP-1-derived macrophages treated with rMS for 3 h prior to *S. aureus* infection, RT-qPCR analysis revealed a significant reduction in the transcriptional expression of IL-1β and TNF-α (Figure 6A). However, no significant change was observed for IL-6 mRNA expression.

Moreover, THP-1-derived macrophages pretreated with rMS for 3 h and subsequently infected with *S. aureus* for an additional 3 h exhibited a decrease in p62 protein expression (Figure 6B). Analysis of confocal images using Image J v1.53k demonstrated a similar decrease in p62 fluorescent intensity in the puncta of cells (Figure 6C). These results suggest the potential involvement of autophagy in the intracellular survival of *S. aureus*.

### 3.7. rMS Decreases S. aureus-Mediated Phosphorylation of p38 MAPK and Its Nuclear Localization

**Figure 7:**
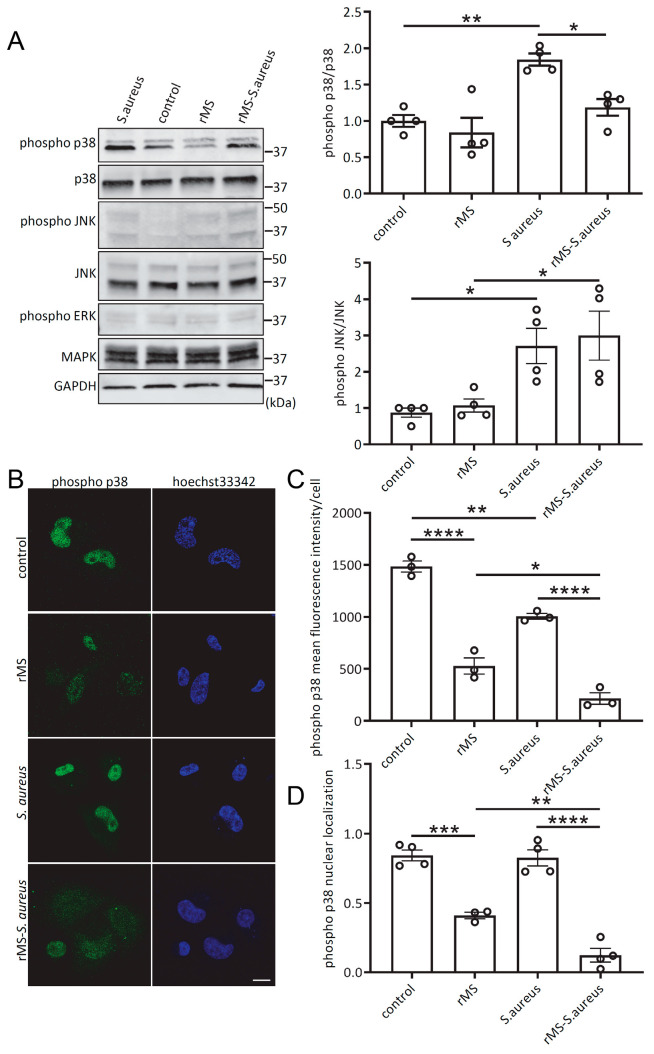
rMS decreases p38 MAPK phosphorylation and nuclear localization. (**A**) THP-1-derived macrophages were treated with rMS 3 h prior to infection with *S. aureus*. Cell lysis was performed 1 h after infection using RIPA supplemented with a protease and phosphatase inhibitors cocktail. The immunoblots shown here are representative of 4 independent experiments. Densitometric analysis was performed using Image Studio Lite. (**B**) Immunofluorescence confocal microscopy. Confocal images of THP-1-derived macrophages treated with rMS and infected with Hoechst-labeled *S. aureus*. (**C**) Quantitative analysis of phospho-p38 fluorescence intensity per cell. (**D**) Quantification of the phospho-p38 localization in the nucleus in THP-1-derived macrophages treated with rMS and infected with *S. aureus*. * *p* < 0.05, ** *p* < 0.01, *** *p* < 0.0005, **** *p* < 0.0001 were determined via one-way ANOVA with Tukey’s multiple comparisons.

Since previous studies have shown that *S. aureus* is able to modulate the JNK MAPK signaling pathway in RAW264.7 macrophages [22], our aim was to investigate the involvement of the MAPK signaling pathway in THP-1-derived macrophages treated with rMS and subsequently infected with *S. aureus*. Protein lysates extracted from THP-1-derived macrophages stimulated with rMS for 3 h and then infected with *S. aureus* for an additional 1 h were analyzed via Western blot. The results showed that rMS treatment alone had no significant effect on the activation of p38, JNK, and ERK MAPK signaling (Figure 7A). In contrast, infection of THP-1-derived macrophages with *S. aureus* strongly induced phosphorylation of p38 and JNK, while ERK phosphorylation remained unchanged. Interestingly, in *S. aureus*-infected cells, rMS treatment specifically inhibited the phosphorylation of p38 compared to control cells.

To further validate the findings from the Western blot analysis, we acquired confocal images of THP-1-derived macrophages labeled with a specific phospho-p38 MAPK antibody, counterstained the nuclei with Hoechst 33342, and conducted quantitative analysis using Image J software v1.53k (Figure 7B). rMS-treated THP-1-derived macrophages exhibited a significant decrease in the fluorescence intensity of phospho-p38 MAPK compared to control cells or *S. aureus*-infected cells (Figure 7C). Additionally, analysis of confocal images revealed a reduction in the nuclear localization of phospho-p38 MAPK in rMS-treated macrophages compared to control macrophages or *S. aureus*-infected macrophages (Figure 7D).

## 4. Discussion

In this study, we demonstrated that the use of a single 5 min session of high-frequency repetitive magnetic stimulation is able to decrease the intracellular load of *S. aureus* in THP-1-derived macrophages and in primary alveolar macrophages. We determined that rMS treatment activated the Nrf2/Keap1 signaling pathway through a non-canonical mechanism. rMS increased p62 protein expression and p62 phosphorylation, which increased its affinity binding to Keap1, leading to the release of Nrf2 and its translocation to the nucleus. We demonstrated that Nrf2 was necessary to the bactericidal activity of macrophages since Nrf2 knockdown, using siRNA in THP-1-derived macrophages or alveolar macrophages isolated from Nrf2 knockout mice, led to an abolition of the rMS-mediated bactericidal activity. Furthermore, we showed that *S. aureus*-induced activation of p38 MAPK was repressed by rMS treatment. rMS-mediated repression of p38 MAPK may explain the decrease in *S. aureus*-induced expression of genes coding for the pro-inflammatory cytokines IL-1β and TNF-α. To the best of our knowledge, this study is the first report to link macrophage biological activity and the molecular mechanisms elicited by rMS treatment.

rMS has become an increasingly popular non-invasive technique for modulating neural excitability and plasticity in clinical and preclinical models, as well as in cultured neurons. However, the effect of rMS on non-excitable cells should not be overlooked. Recent reports have described the significant role of rMS in various cell types, including microglia, mesenchymal stromal cells, chondrocytes, and endothelial cells, despite the discrepancies in the stimulatory parameters, which include variations in intensity, frequency, and duration [18,19,23,24,25].

One recent report conducted on lipopolysaccharide-stimulated microglia isolated from newborn Wistar rat brains and treated twice with 10 Hz rTMS showed no effect of rTMS on IL-1β and TNF-α secretions while increasing the production of IL-4 and IL-10. The authors stated that rTMS treatment could promote the anti-inflammatory polarization of microglia [25]. Similarly, another recent study demonstrated the role played by microglia in inducing synaptic plasticity through the release of cytokines upon rTMS treatment in a mouse model of organotypic brain tissue. Furthermore, in vivo depletion of microglia abolished rTMS-induced synaptic plasticity [18]. In our study, we showed that a 5 min rMS treatment of THP-1-derived macrophages did not significantly alter transcriptional expression levels of IL-6, IL-1β, and TNF-α. However, when THP-1-derived macrophages were pretreated with rMS prior to *S. aureus* infection, a decrease in the transcriptional expression levels of genes coding for IL-1β and TNF-β was observed. While the effects of rMS may vary depending on the specific cell type and variations in stimulatory parameters such as intensity, duration and frequency of treatment, it is reasonable to postulate that rMS may have a significant effect on the inflammatory processes of innate immune cells. Therefore, considering the diverse and still largely unexplored impact of rMS on cell–cell and cell–microenvironment interactions, we have decided to investigate the cellular and molecular responses induced by rMS in an in vitro model of *S. aureus*-infected macrophages.

We have previously demonstrated that the anti-inflammatory effect of Nrf2 signaling pathway in our in vitro model of *S. aureus*-infected THP-1-derived macrophages was obtained through repression of p38 and JNK MAPK phosphorylation. *S. aureus*-mediated activation of p38 and JNK MAPK signaling pathways was inhibited by the pretreatment of THP-1-derived macrophages with sulforaphane, a well-established Nrf2 activator. Repression of p38 and JNK MAPK phosphorylation led to a significant decrease in transcriptional expression levels of pro-inflammatory cytokines [5]. In the present study, we observed an increase in the phosphorylation of p38 and JNK MAPK in THP-1-derived macrophages infected with *S. aureus*. rMS treatment of macrophages specifically repressed *S. aureus*-induced p38 MAPK phosphorylation and decreased phosphorylated p38 nuclear localization, while showing no effect of rMS on *S. aureus*-mediated activation of JNK MAPK.

Interestingly, intracellular bacterial pathogens have evolved various strategies to escape degradation mechanisms. Early activation of the p38 MAPK signaling pathway is one of the strategies employed by *S. aureus* to survive in the host cell [26]. Indeed, activation of p38 MAPK by *S. aureus* has been found to block autophagy degradation and ensure bacterial survival within the host cell [27]. Specifically, it has been shown that p38 MAPK can activate the NF-κB, which in turn promotes the expression of p62.

rMS treatment of THP-1-derived macrophages resulted in the activation of the Nrf2 signaling pathway, accompanied by a decrease in Keap1 protein level. The lack of any change in oxidative stress observed in rMS-stimulated macrophages suggests that the activation of Nrf2 by rMS does not occur through the canonical mechanism. Instead, our data indicate that rMS activates Nrf2 via a non-canonical mechanism involving the upregulation of the autophagy-adaptor protein p62 and its subsequent phosphorylation. Phosphorylation of p62 enhances its binding affinity to Keap1, resulting in Nrf2 accumulation and facilitating its translocation to the nucleus [21]. The phosphorylated p62-Keap1 complex leads to autophagic degradation. Notably, Nrf2 also positively regulates the transcriptional expression of p62 due to the presence of ARE sequences in the p62 promoter, establishing a positive feedback loop [28].

The molecular mechanism responsible for the activation of p62 in response to rMS treatment in our in vitro cell model remains unclear. Previous studies have suggested that mammalian target of rapamycin complex 1 (mTORC1) may be involved in the phosphorylation of the Ser349 residue, which is located within the KIR domain of p62 and implicated in Keap1 affinity binding [21]. Therefore, it would be valuable to explore whether the modulation of p62 by rMS is dependent on mTORC1 activity. Furthermore, studies conducted on differentiated Neuro-2a cells have shown that high-frequency rMS treatment led to the activation of Ca^2+^-calmodulin-dependent protein kinase-cAMP-response element binding protein (CREB) signaling pathway, resulting in increased brain-derived neurotrophic factor (BDNF) expression and synaptic plasticity. Moreover, rMS has been reported to reduce neuronal cell death and promote cell proliferation while inhibiting apoptosis through activation of the ERK and AKT-signaling pathways [29]. In addition, bone mesenchymal stromal cells stimulated with high-frequency rTMS demonstrated autophagy flux through the activation of the NMDAR-Ca^2+^-ERK-mTOR signaling pathway [19].

Autophagy is a fundamental biological process against *S. aureus* and has been shown to play a crucial role in the control of *S. aureus* growth. Previous studies have described a recruitment of p62 to *S. aureus* in vitro in fibroblasts and epithelial cells [27]. Also, a recent report has revealed the interaction of p62 with *S. aureus* in vivo in neutrophils of zebrafish [30]. The disruption of p62 expression in neutrophils resulted in reduced zebrafish survival after *S. aureus* infection. Since both the p62 and p38 MAPK pathways are well-known players in autophagy, and here are involved in the bactericidal activity of rMS-treated macrophages, it would be interesting to investigate the potential role of autophagy in modulating macrophage function against pathogens via rMS. Overall, these findings indicate a complex interplay between Nrf2/Keap1/p62, *S. aureus*, and the p38 MAPK pathway in response to rMS treatment. Therefore, it would be interesting to investigate the potential impact of rMS on autophagy. While the effects of rMS have been widely acknowledged in clinical and preclinical models, it remains a challenge to apply rMS on systemic immune cells and elicit specific responses due to the limited area targeted by magnetic stimulation. One potential therapeutic strategy to consider would involve the promising developments in ex vivo-activated macrophage-based therapies [31].

## 5. Conclusions

**Figure 8:**
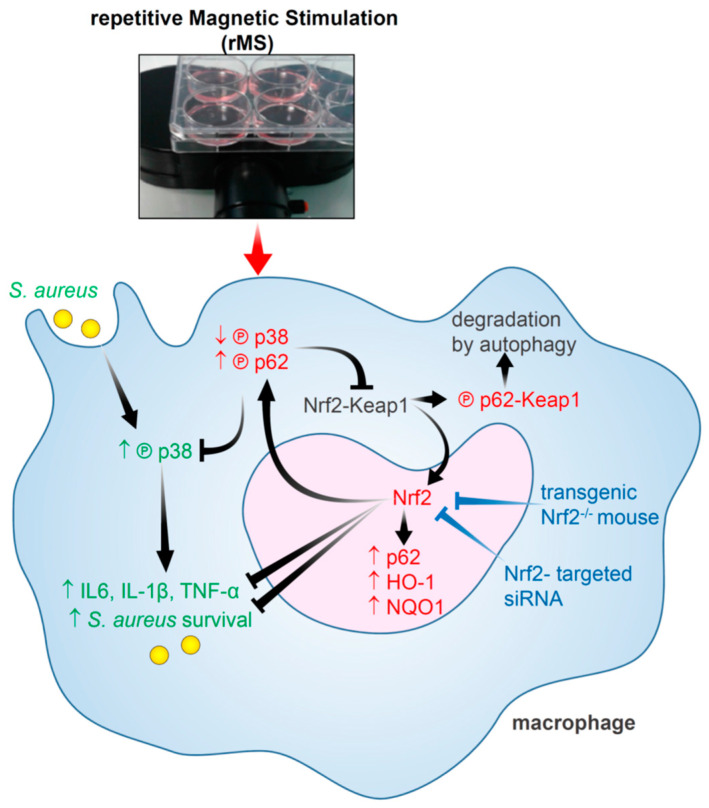
Schematic representation of the p62/Keap1/Nrf2 non-canonical pathway activated by rMS in THP-1-derived macrophages infected with *S. aureus*. THP-1-derived macrophages infected with *S. aureus* mediate an activation of the p38 MAPK pathway, which regulates the gene expression of pro-inflammatory cytokines such as IL-6, IL-1β, and TNF-α, and promote *S. aureus* intracellular survival. In THP-1-derived macrophages, treatment with rMS results in an increase in phosphorylated p62 at Ser349, which results in increased interaction with Keap1 and the release of Nrf2. The phosphorylated p62-Keap1 complex is identified for degradation via autophagy. Nrf2 is then free to translocate to the nucleus, interact with small Maf proteins, and bind antioxidant response element (ARE), leading to the upregulation of genes coding for antioxidant, detoxifying, and anti-inflammatory proteins, as well as coding for p62. Nrf2 silencing was obtained by utilizing specific siRNA in THP-1-derived macrophages or utilizing alveolar macrophages isolated from Nrf2 knockout mice.

In summary, we propose a molecular signaling pathway activated by rMS in THP-1-derived macrophages based on our findings (Figure 8). rMS treatment activates the Nrf2 signaling pathway through the Nrf2/Keap1/p62 pathway. The p62/Keap1 complex undergoes autophagy degradation, while unbound Nrf2 translocates to the nucleus and promotes the transcriptional upregulation of genes involved in detoxification, antioxidant defense, and anti-inflammatory mechanisms. In addition, rMS treatment inhibits the p38 MAPK signaling pathway, which is implicated in *S. aureus* intracellular survival and pro-inflammatory responses.

## Figures and Tables

**Table 1:** List of human primer sequences for RT-qPCR.

Gene	Forward Primer	Reverse Primer	Gene Accession Code
Nrf2	5′-AGCGACGGAAAGAGTATGAG-3′	5′-GTTGGCAGATCCACTGGTTT-3′	NM_001145413.2
HO-1	5′-TCCGATGGGTCCTTACACTC-3′	5′-TAAGGAAGCCAGCCAAGAGA-3′	NM_002133.2
NQO1	5′-CAGACGCCCGAATTCAAATC-3′	5′-AGGCTGCTTGGAGCAAAATACA-3′	NM_000903.2
IL-1β	5′-AATGATGGCTTATTACAGTGGCA-3′	5′-GTCGGAGATTCGTAGCTGGA-3′	NM_000576.3
IL-6	5′-GTAGCCGCCCCACACAGA-3′	5′-CATGTCTCCTTTCTCAGGGCTG-3′	NM_000600.5
TNF-α	5′-GGAGAAGGGTGACCGACTC-3′	5′-TGGGAAGGTTGGATGTTCGT-3′	NM_000594
18S rRNA	5′-GATAGCTCTTTCTCGATTCCG-3′	5′-TAGTTAGCATGCCAGAGTC-3′	M10098.1

## Data Availability

All the data are contained within the article and the Supplementary Figure S1.

## Supplementary Materials

The following supporting information can be downloaded at: https://www.mdpi.com/article/10.3390/antiox12091695/s1, Figure S1: THP-1-derived macrophages were subjected to rMS protocol 5 Hz and 15 Hz and cells were lysed at the indicated time.

## Abbreviations

cDNA: complementary deoxyribonucleic acid; CFU: colony-forming unit; DAPI: 4′, 6-diamidino-2-phenylindole; DMSO: dimethyl sulfoxide; ERK: extracellular signal-regulated kinase; HO-1: Heme oxygenase-1; IL-1β: interleukin-1β; IL-6: interleukin-6; JNK: c-Jun N-terminal kinase; Keap1: Kelch-like ECH-associated protein 1; Maf: musculoaponeurotic fibrosarcoma; MOI: multiplicity of infection; MTT: 3-(4,5-dimethylthiazol-2-yl)-2,5-diphenyltetrazolium bromide; NQO1: NAD(P)H quinone dehydrogenase 1; Nrf2/NFE2L2: nuclear factor erythroid 2-related factor 2; p38 MAPK: p38 mitogen-activated protein kinase; p62/SQSTM1: sequestosome 1; PBS: phosphate-buffered saline; PCR: polymerase chain reaction; PFA: paraformaldehyde; RNA: ribonucleic acid; RIPA: radioimmunoprecipitation assay buffer; rMS: repetitive magnetic stimulation; ROS: reactive oxygen species; rTMS: repetitive transcranial magnetic stimulation; siRNA: small interfering RNA; THP-1: human leukemic cell line; TNF-α: tumor necrosis factor-α.

## References

[1] Lazarov T., Juarez-Carreno S., Cox N., Geissmann F. (2023). Physiology and Diseases of Tissue-Resident Macrophages. Nature.

[2] DeLeo F.R., Otto M., Kreiswirth B.N., Chambers H.F. (2010). Community-Associated Meticillin-Resistant *Staphylococcus aureus*. Lancet.

[3] Martin F.J., Parker D., Harfenist B.S., Soong G., Prince A. (2011). Participation of Cd11c(+) Leukocytes in Methicillin-Resistant *Staphylococcus aureus* Clearance from the Lung. Infect. Immun..

[4] Nelson R.E., Hyun D., Jezek A., Samore M.H. (2022). Mortality, Length of Stay, and Healthcare Costs Associated with Multidrug-Resistant Bacterial Infections among Elderly Hospitalized Patients in the United States. Clin. Infect. Dis..

[5] Deramaudt T.B., Ali M., Vinit S., Bonay M. (2020). Sulforaphane Reduces Intracellular Survival of *Staphylococcus aureus* in Macrophages through Inhibition of Jnk and P38 Mapk-Induced Inflammation. Int. J. Mol. Med..

[6] Ellington J.K., Elhofy A., Bost K.L., Hudson M.C. (2001). Involvement of Mitogen-Activated Protein Kinase Pathways in *Staphylococcus aureus* Invasion of Normal Osteoblasts. Infect. Immun..

[7] Gomez M.I., Lee A., Reddy B., Muir A., Soong G., Pitt A., Cheung A., Prince A. (2004). *Staphylococcus aureus* Protein a Induces Airway Epithelial Inflammatory Responses by Activating Tnfr1. Nat. Med..

[8] Kujime K., Hashimoto S., Gon Y., Shimizu K., Horie T. (2000). P38 Mitogen-Activated Protein Kinase and C-Jun-Nh2-Terminal Kinase Regulate Rantes Production by Influenza Virus-Infected Human Bronchial Epithelial Cells. J. Immunol..

[9] Bonay M., Roux A.L., Floquet J., Retory Y., Herrmann J.L., Lofaso F., Deramaudt T.B. (2015). Caspase-Independent Apoptosis in Infected Macrophages Triggered by Sulforaphane Via Nrf2/P38 Signaling Pathways. Cell Death Discov..

[10] Boutten A., Goven D., Artaud-Macari E., Boczkowski J., Bonay M. (2011). Nrf2 Targeting: A Promising Therapeutic Strategy in Chronic Obstructive Pulmonary Disease. Trends Mol. Med..

[11] Silva-Islas C.A., Maldonado P.D. (2018). Canonical and Non-Canonical Mechanisms of Nrf2 Activation. Pharmacol. Res..

[12] Komatsu M., Kurokawa H., Waguri S., Taguchi K., Kobayashi A., Ichimura Y., Sou Y.S., Ueno I., Sakamoto A., Tong K.I. (2010). The Selective Autophagy Substrate P62 Activates the Stress Responsive Transcription Factor Nrf2 through Inactivation of Keap1. Nat. Cell Biol..

[13] Hast B.E., Goldfarb D., Mulvaney K.M., Hast M.A., Siesser P.F., Yan F., Hayes D.N., Major M.B. (2013). Proteomic Analysis of Ubiquitin Ligase Keap1 Reveals Associated Proteins That Inhibit Nrf2 Ubiquitination. Cancer Res..

[14] Karapetian R.N., Evstafieva A.G., Abaeva I.S., Chichkova N.V., Filonov G.S., Rubtsov Y.P., Sukhacheva E.A., Melnikov S.V., Schneider U., Wanker E.E. (2005). Nuclear Oncoprotein Prothymosin Alpha Is a Partner of Keap1: Implications for Expression of Oxidative Stress-Protecting Genes. Mol. Cell. Biol..

[15] Ma J., Cai H., Wu T., Sobhian B., Huo Y., Alcivar A., Mehta M., Cheung K.L., Ganesan S., Kong A.N. (2012). Palb2 Interacts with Keap1 to Promote Nrf2 Nuclear Accumulation and Function. Mol. Cell. Biol..

[16] Vinhas A., Almeida A.F., Goncalves A.I., Rodrigues M.T., Gomes M.E. (2020). Magnetic Stimulation Drives Macrophage Polarization in Cell to-Cell Communication with Il-1beta Primed Tendon Cells. Int. J. Mol. Sci..

[17] Somaa F.A., de Graaf T.A., Sack A.T. (2022). Transcranial Magnetic Stimulation in the Treatment of Neurological Diseases. Front. Neurol..

[18] Eichler A., Kleidonas D., Turi Z., Fliegauf M., Kirsch M., Pfeifer D., Masuda T., Prinz M., Lenz M., Vlachos A. (2023). Microglial Cytokines Mediate Plasticity Induced by 10 Hz Repetitive Magnetic Stimulation. J. Neurosci..

[19] Wang X., Zhou X., Bao J., Chen Z., Tang J., Gong X., Ni J., Fang Q., Liu Y., Su M. (2019). High-Frequency Repetitive Transcranial Magnetic Stimulation Mediates Autophagy Flux in Human Bone Mesenchymal Stromal Cells Via Nmda Receptor-Ca(2+)-Extracellular Signal-Regulated Kinase-Mammalian Target of Rapamycin Signaling. Front. Neurosci..

[20] Itoh K., Mochizuki M., Ishii Y., Ishii T., Shibata T., Kawamoto Y., Kelly V., Sekizawa K., Uchida K., Yamamoto M. (2004). Transcription Factor Nrf2 Regulates Inflammation by Mediating the Effect of 15-Deoxy-Delta(12,14)-Prostaglandin J(2). Mol. Cell. Biol..

[21] Ichimura Y., Waguri S., Sou Y.S., Kageyama S., Hasegawa J., Ishimura R., Saito T., Yang Y., Kouno T., Fukutomi T. (2013). Phosphorylation of P62 Activates the Keap1-Nrf2 Pathway During Selective Autophagy. Mol. Cell.

[22] Fang L., Wu H.M., Ding P.S., Liu R.Y. (2014). Tlr2 Mediates Phagocytosis and Autophagy through Jnk Signaling Pathway in *Staphylococcus aureus*-Stimulated Raw264.7 Cells. Cell. Signal.

[23] Zong X., Li Y., Liu C., Qi W., Han D., Tucker L., Dong Y., Hu S., Yan X., Zhang Q. (2020). Theta-Burst Transcranial Magnetic Stimulation Promotes Stroke Recovery by Vascular Protection and Neovascularization. Theranostics.

[24] Elliott J.P., Smith R.L., Block C.A. (1988). Time-Varying Magnetic Fields: Effects of Orientation on Chondrocyte Proliferation. J. Orthop. Res..

[25] Luo J., Feng Y., Li M., Yin M., Qin F., Hu X. (2022). Repetitive Transcranial Magnetic Stimulation Improves Neurological Function and Promotes the Anti-Inflammatory Polarization of Microglia in Ischemic Rats. Front. Cell. Neurosci..

[26] Miller M., Dreisbach A., Otto A., Becher D., Bernhardt J., Hecker M., Peppelenbosch M.P., van Dijl J.M. (2011). Mapping of Interactions between Human Macrophages and *Staphylococcus aureus* Reveals an Involvement of Map Kinase Signaling in the Host Defense. J. Proteome Res..

[27] Neumann Y., Bruns S.A., Rohde M., Prajsnar T.K., Foster S.J., Schmitz I. (2016). Intracellular *Staphylococcus aureus* Eludes Selective Autophagy by Activating a Host Cell Kinase. Autophagy.

[28] Jain A., Lamark T., Sjottem E., Larsen K.B., Awuh J.A., Overvatn A., McMahon M., Hayes J.D., Johansen T. (2010). P62/Sqstm1 Is a Target Gene for Transcription Factor Nrf2 and Creates a Positive Feedback Loop by Inducing Antioxidant Response Element-Driven Gene Transcription. J. Biol. Chem..

[29] Baek A., Kim J.H., Pyo S., Jung J.H., Park E.J., Kim S.H., Cho S.R. (2018). The Differential Effects of Repetitive Magnetic Stimulation in an in Vitro Neuronal Model of Ischemia/Reperfusion Injury. Front. Neurol..

[30] Gibson J.F., Prajsnar T.K., Hill C.J., Tooke A.K., Serba J.J., Tonge R.D., Foster S.J., Grierson A.J., Ingham P.W., Renshaw S.A. (2021). Neutrophils Use Selective Autophagy Receptor Sqstm1/P62 to Target *Staphylococcus aureus* for Degradation in Vivo in Zebrafish. Autophagy.

[31] Mishra A.K., Malonia S.K. (2023). Advancing Cellular Immunotherapy with Macrophages. Life Sci..

