# Supplementary material for: High-Frequency Repetitive Magnetic Stimulation Activates Bactericidal Activity of Macrophages via Modulation of p62/Keap1/Nrf2 and p38 MAPK Pathways
Source: Antioxidants (Basel). 2023 Aug 30;12(9):1695. doi: 10.3390/antiox12091695 (PMC10525279; doi:10.3390/antiox12091695)
Supplement: Supplementary file 1 [file antioxidants-12-01695-s001.zip › antioxidants-2544756-supplementary (1).pdf]

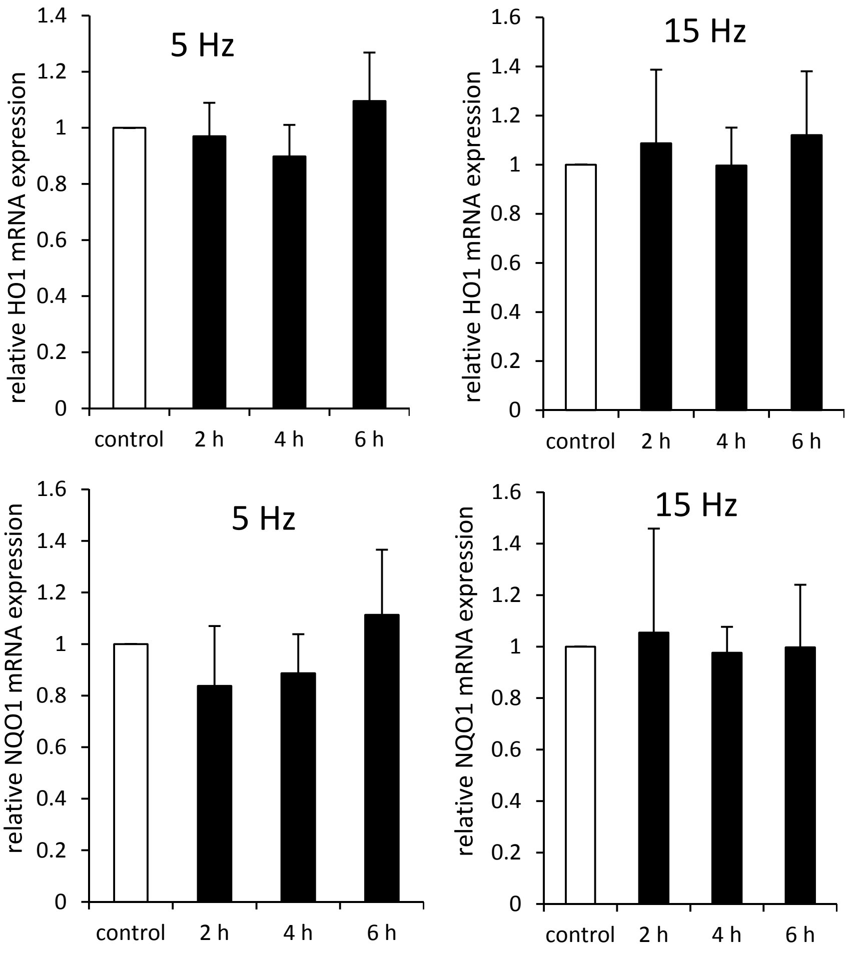


**Figure S1.** THP-1-derived macrophages were subjected to rMS protocol 5 Hz and 15 Hz and cells were lysed at the indicated time.
