# Supplementary material for: High-Frequency Repetitive Magnetic Stimulation Activates Bactericidal Activity of Macrophages via Modulation of p62/Keap1/Nrf2 and p38 MAPK Pathways
Source: Antioxidants (Basel). 2023 Aug 30;12(9):1695. doi: 10.3390/antiox12091695 (PMC10525279; doi:10.3390/antiox12091695)
Supplement: Supplementary file 1 [file antioxidants-12-01695-s001.zip › antioxidants-2544756-supplementary.pptx]

## Slide 1
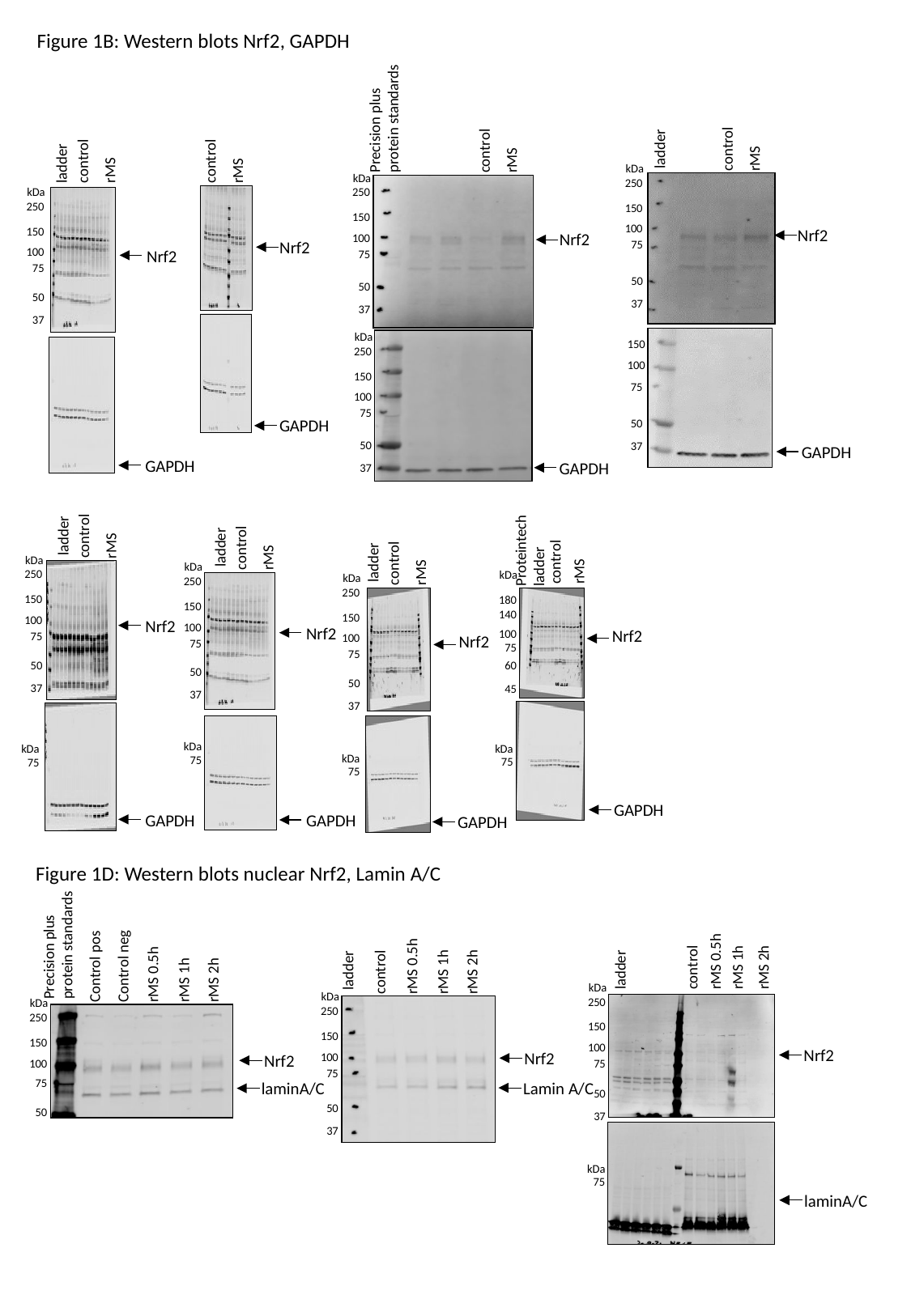

Figure 1B: Western blots Nrf2, GAPDH
Precision plus protein standards
ladder
control
control
rMS
rMS
control
control
ladder
kDa
rMS
rMS
kDa
250
kDa
250
250
150
150
100
150
Nrf2
Nrf2
100
Nrf2
75
100
Nrf2
75
75
50
50
50
37
37
37
kDa
150
250
100
150
75
100
75
GAPDH
50
50
37
GAPDH
GAPDH
GAPDH
37
ladder
control
Proteintech
ladder
rMS
ladder
control
rMS
kDa
control
ladder
control
kDa
rMS
rMS
250
kDa
kDa
250
250
150
180
150
140
150
100
Nrf2
100
Nrf2
Nrf2
100
75
100
Nrf2
75
75
75
50
60
50
50
37
45
37
37
kDa
kDa
kDa
kDa
75
75
75
75
GAPDH
GAPDH
GAPDH
GAPDH
Figure 1D: Western blots nuclear Nrf2, Lamin A/C
Precision plus protein standards
rMS 0.5h
Control neg
rMS 0.5h
Control pos
control
rMS 1h
rMS 2h
ladder
ladder
control
rMS 1h
rMS 2h
rMS 0.5h
rMS 1h
rMS 2h
kDa
kDa
250
kDa
250
250
150
150
150
100
Nrf2
Nrf2
100
Nrf2
100
75
75
75
Lamin A/C
laminA/C
50
50
50
37
37
kDa
75
laminA/C

## Slide 2
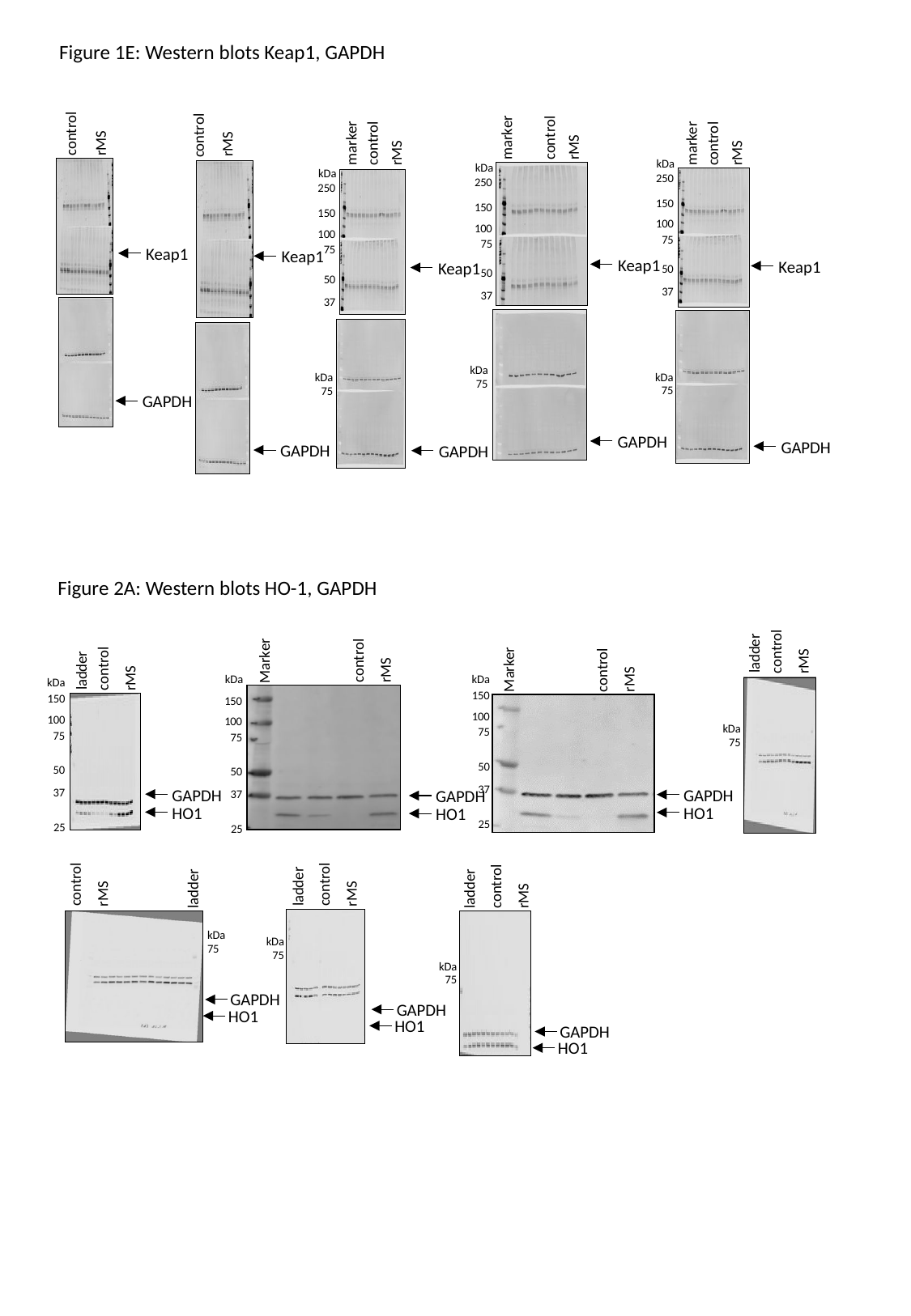

Figure 1E: Western blots Keap1, GAPDH
control
control
marker
control
marker
control
rMS
marker
control
rMS
rMS
rMS
rMS
kDa
kDa
kDa
250
250
250
150
150
150
100
100
100
75
75
75
Keap1
Keap1
Keap1
Keap1
Keap1
50
50
50
37
37
37
kDa
kDa
kDa
75
75
75
GAPDH
GAPDH
GAPDH
GAPDH
GAPDH
Figure 2A: Western blots HO-1, GAPDH
control
ladder
control
Marker
rMS
control
Marker
control
rMS
ladder
rMS
rMS
kDa
kDa
kDa
150
150
150
100
100
100
kDa
75
75
75
75
50
50
50
37
GAPDH
GAPDH
37
GAPDH
37
HO1
HO1
HO1
25
25
25
control
control
control
ladder
ladder
ladder
rMS
rMS
rMS
kDa
kDa
75
75
kDa
75
GAPDH
GAPDH
HO1
HO1
GAPDH
HO1

## Slide 3
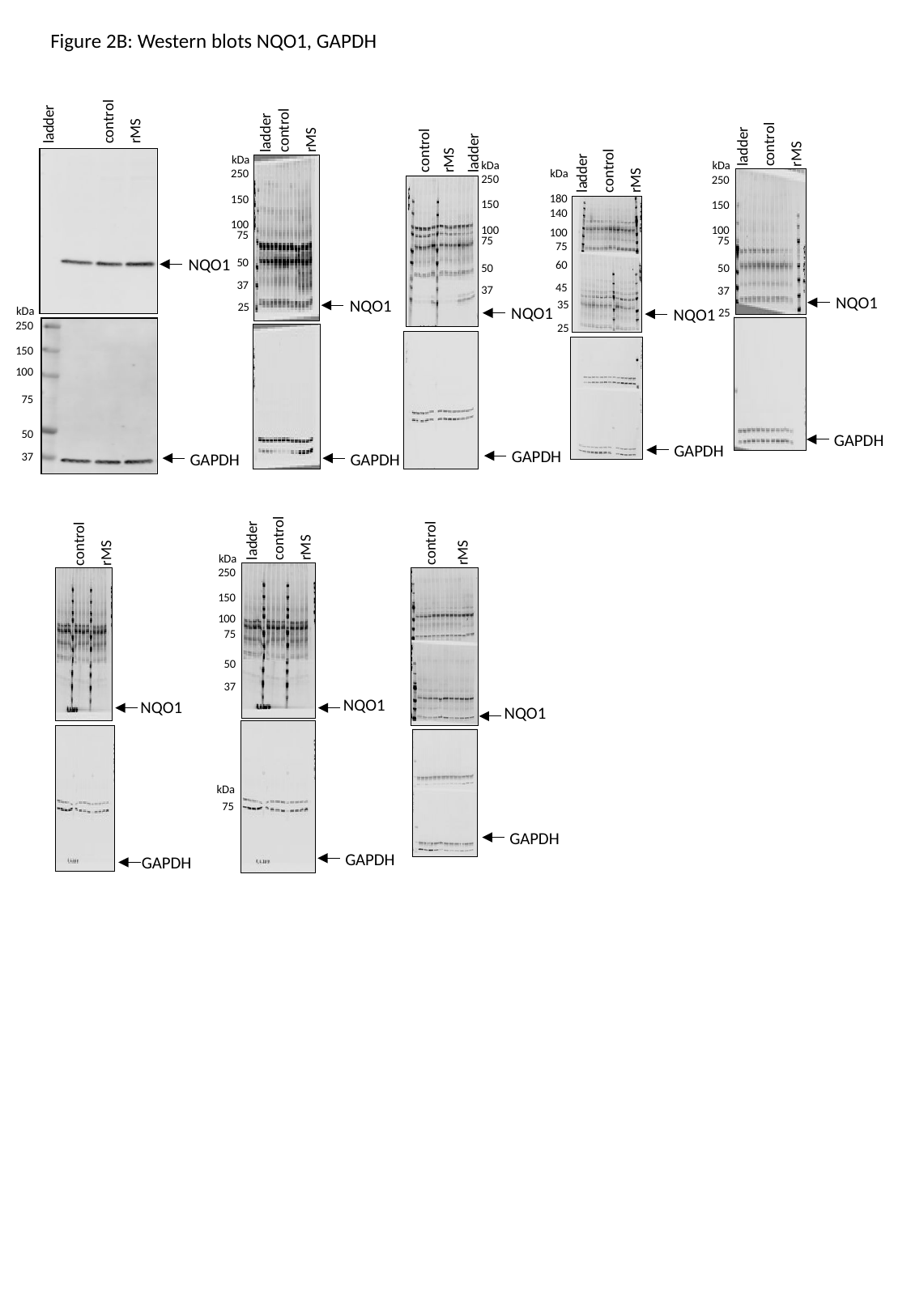

Figure 2B: Western blots NQO1, GAPDH
control
ladder
control
rMS
ladder
rMS
control
ladder
control
ladder
rMS
rMS
kDa
kDa
kDa
control
ladder
250
kDa
rMS
250
250
180
150
150
150
140
100
100
100
100
75
75
75
75
NQO1
50
60
50
50
37
45
37
37
NQO1
NQO1
35
25
NQO1
kDa
NQO1
25
250
25
150
100
75
50
GAPDH
GAPDH
GAPDH
GAPDH
GAPDH
37
control
ladder
control
control
rMS
rMS
rMS
kDa
250
150
100
75
50
37
NQO1
NQO1
NQO1
kDa
75
GAPDH
GAPDH
GAPDH

## Slide 4
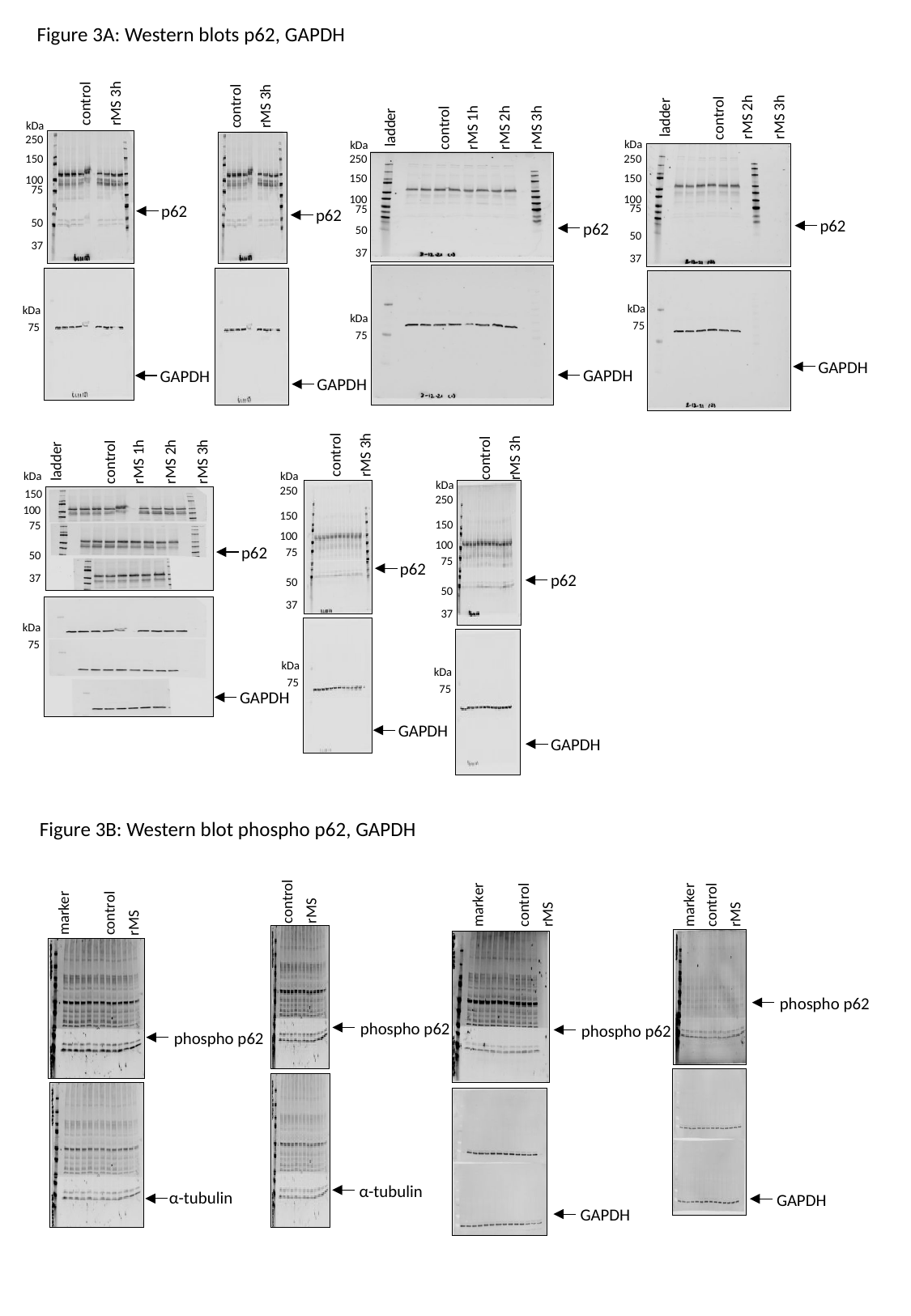

Figure 3A: Western blots p62, GAPDH
control
rMS 3h
control
rMS 3h
rMS 2h
rMS 3h
ladder
control
kDa
ladder
control
rMS 1h
rMS 2h
rMS 3h
250
kDa
kDa
250
150
250
150
150
100
75
100
100
p62
75
75
p62
50
p62
p62
50
50
37
37
37
kDa
kDa
kDa
75
75
75
GAPDH
GAPDH
GAPDH
GAPDH
control
rMS 3h
control
rMS 3h
ladder
control
rMS 1h
rMS 2h
rMS 3h
kDa
kDa
kDa
250
150
250
100
150
150
75
100
100
p62
75
50
75
p62
p62
37
50
50
37
37
kDa
75
kDa
kDa
75
75
GAPDH
GAPDH
GAPDH
Figure 3B: Western blot phospho p62, GAPDH
control
marker
marker
control
control
rMS
marker
control
rMS
rMS
rMS
phospho p62
phospho p62
phospho p62
phospho p62
α-tubulin
α-tubulin
GAPDH
GAPDH

## Slide 5
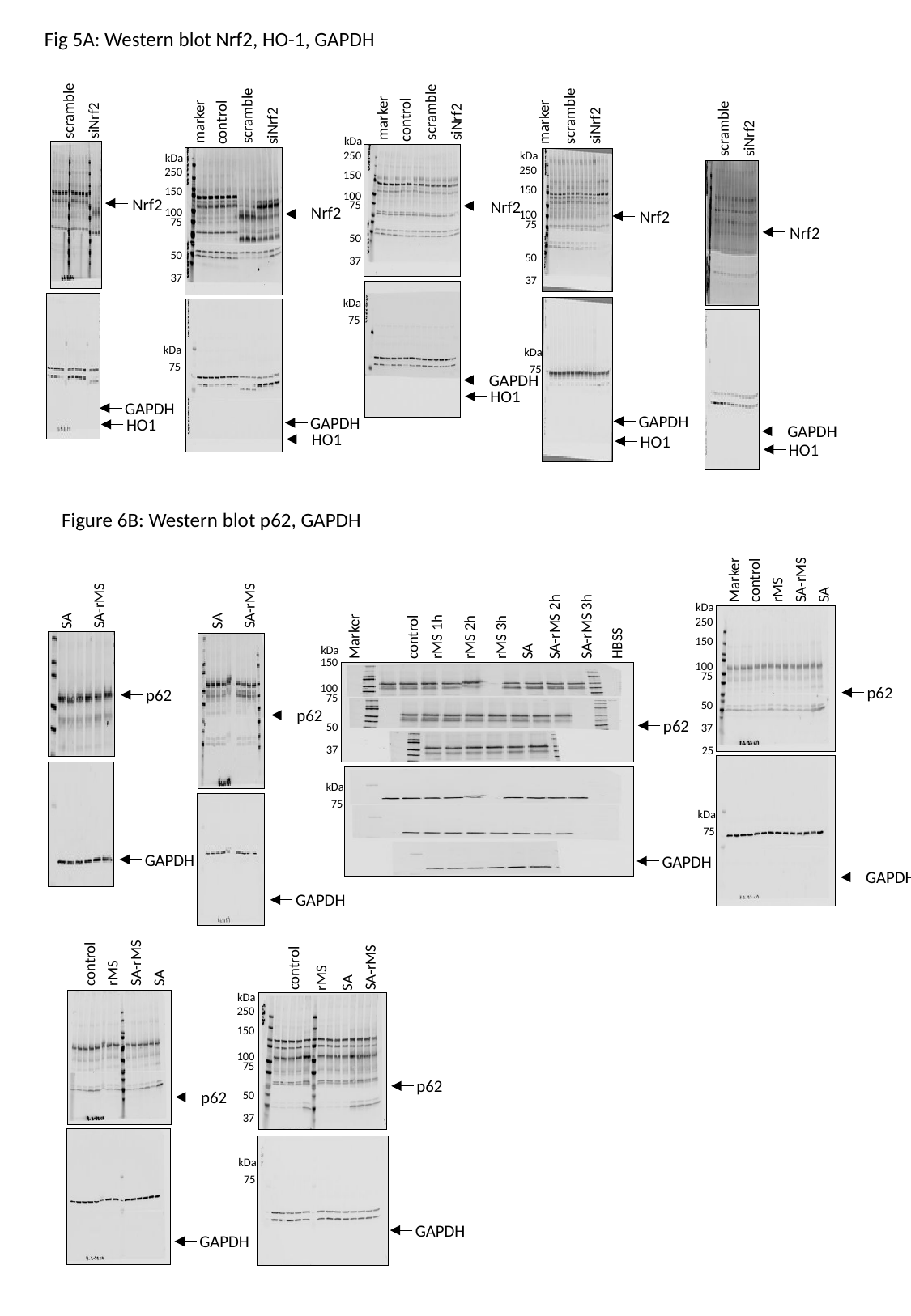

Fig 5A: Western blot Nrf2, HO-1, GAPDH
scramble
scramble
scramble
scramble
marker
control
siNrf2
marker
siNrf2
marker
control
siNrf2
siNrf2
scramble
siNrf2
kDa
kDa
250
kDa
250
250
150
150
150
100
Nrf2
Nrf2
75
Nrf2
100
Nrf2
100
75
75
Nrf2
50
50
50
37
37
37
kDa
75
kDa
kDa
75
75
GAPDH
HO1
GAPDH
GAPDH
GAPDH
HO1
GAPDH
HO1
HO1
HO1
Figure 6B: Western blot p62, GAPDH
SA-rMS
Marker
control
rMS
SA
SA-rMS
SA-rMS
kDa
SA
SA
250
SA-rMS 2h
SA-rMS 3h
Marker
control
rMS 1h
rMS 2h
rMS 3h
150
HBSS
SA
kDa
150
100
75
100
p62
p62
75
50
p62
p62
50
37
37
25
kDa
75
kDa
75
GAPDH
GAPDH
GAPDH
GAPDH
SA-rMS
control
SA-rMS
control
rMS
rMS
SA
SA
kDa
250
150
100
75
p62
p62
50
37
kDa
75
GAPDH
GAPDH

## Slide 6
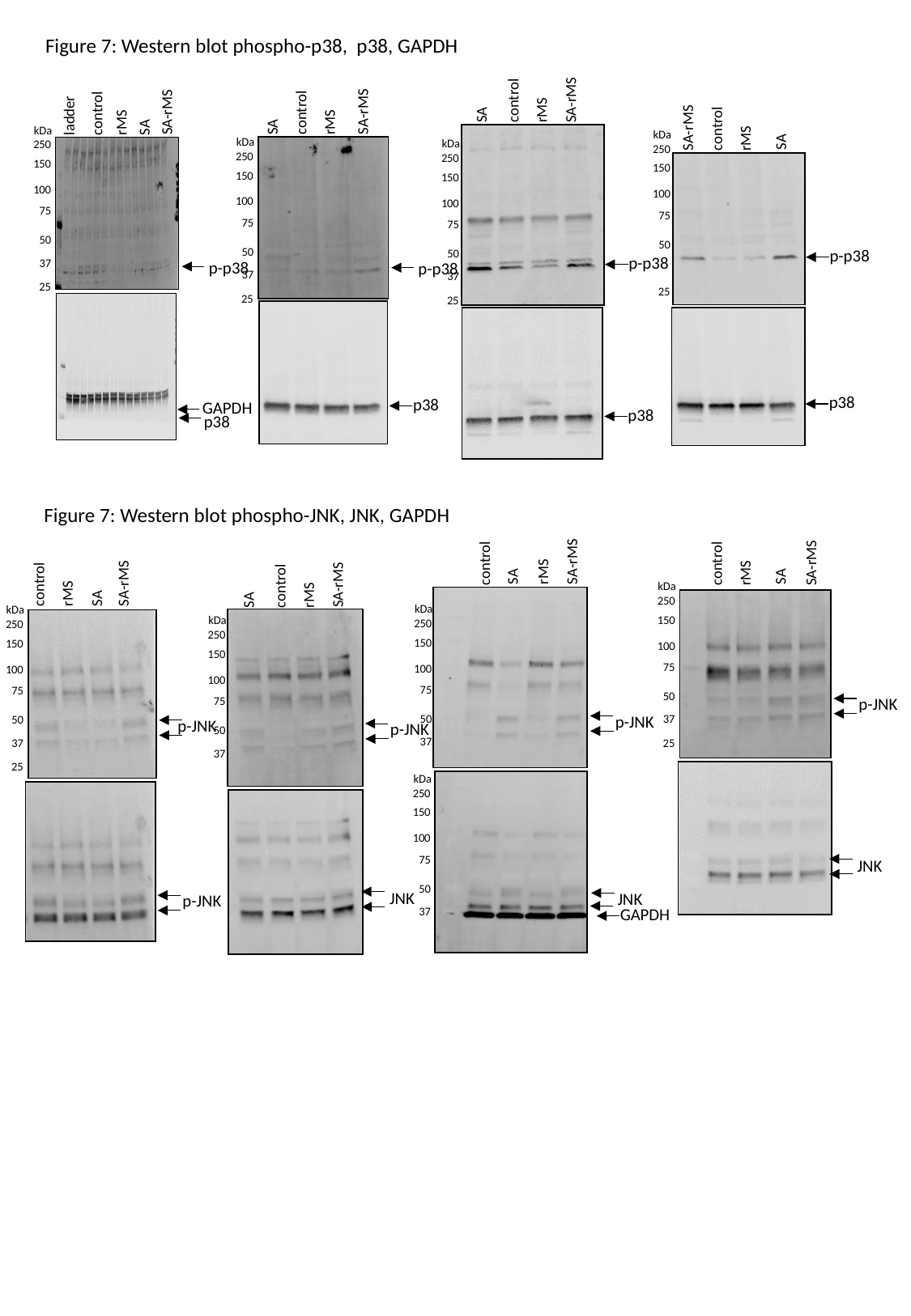

Figure 7: Western blot phospho-p38, p38, GAPDH
SA-rMS
control
rMS
SA-rMS
SA-rMS
control
control
SA
ladder
rMS
rMS
SA
SA
SA-rMS
control
kDa
kDa
rMS
SA
kDa
kDa
250
250
250
250
150
150
150
150
100
100
100
100
75
75
75
75
50
50
50
p-p38
50
p-p38
37
p-p38
p-p38
37
37
25
25
25
25
p38
p38
GAPDH
p38
p38
Figure 7: Western blot phospho-JNK, JNK, GAPDH
SA-rMS
SA-rMS
control
control
rMS
rMS
SA
SA
SA-rMS
control
SA-rMS
control
kDa
rMS
rMS
SA
SA
250
kDa
kDa
150
kDa
250
250
250
150
150
100
150
75
100
100
100
75
75
50
p-JNK
75
p-JNK
50
37
50
p-JNK
p-JNK
50
37
37
25
37
25
kDa
250
150
100
75
JNK
50
JNK
JNK
p-JNK
GAPDH
37

## Slide 7
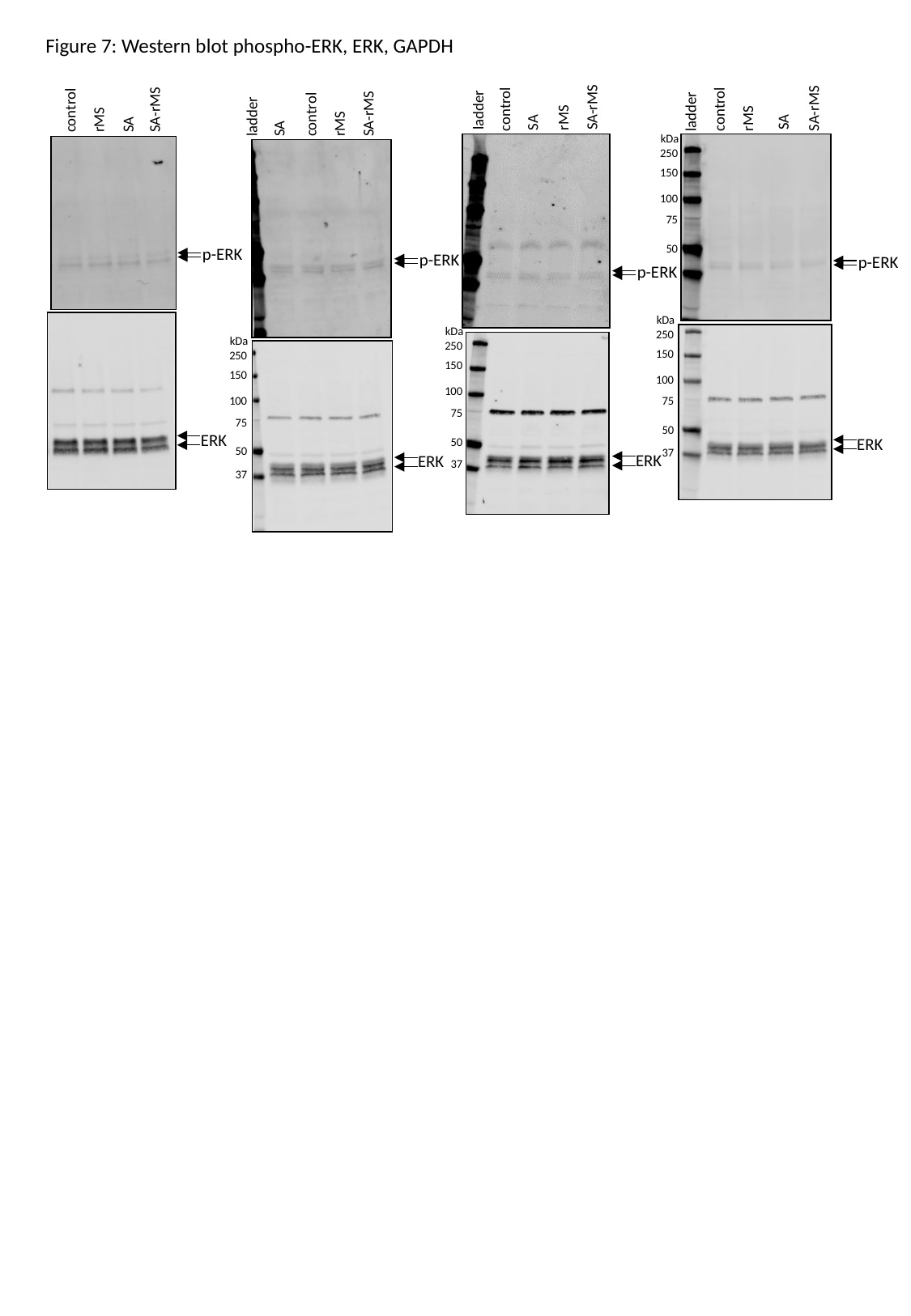

Figure 7: Western blot phospho-ERK, ERK, GAPDH
SA-rMS
SA-rMS
control
control
SA-rMS
ladder
control
ladder
SA-rMS
control
ladder
rMS
rMS
rMS
SA
SA
rMS
SA
SA
kDa
250
150
100
75
50
p-ERK
p-ERK
p-ERK
p-ERK
kDa
kDa
250
kDa
250
150
250
150
150
100
100
100
75
75
75
50
ERK
ERK
50
50
37
ERK
ERK
37
37
